# Signature-based repurposed drugs resemble the inhibition of TGFβ-induced NDRG1 as potential therapeutics for triple-negative breast cancer

**DOI:** 10.7150/ijbs.112645

**Published:** 2025-06-09

**Authors:** Araceli López-Tejada, Jose L. Blaya-Cánovas, Francisca E. Cara, Jesús Calahorra, César Ramírez-Tortosa, Isabel Blancas, Violeta Delgado-Almenta, Fabiola Muñoz-Parra, Marta Ávalos-Moreno, Ana Sánchez, Adrián González-González, Juan A. Marchal, Carmen Griñán-Lisón, Sergio Granados-Principal

**Affiliations:** 1 Department of Biochemistry and Molecular Biology II, Faculty of Pharmacy, University of Granada, Campus de Cartuja s/n, Granada, Spain.; 2 GENYO, Centre for Genomics and Oncological Research, Pfizer/University of Granada/Andalusian Regional Government, Granada, Spain.; 3 Instituto de Investigación Biosanitaria ibs.GRANADA, Granada, Spain.; 4 UGC de Oncología Médica, Hospital Universitario de Jaén, Jaén, Spain.; 5 UGC de Anatomía Patológica, Hospital Universitario "San Cecilio", Granada, Spain.; 6 UGC de Oncología, Hospital Universitario "San Cecilio", Granada, Spain.; 7 Department of Medicine, University of Granada, Granada, Spain.; 8 UGC de Radiodiagnóstico, Hospital Universitario "San Cecilio", Granada, Spain.; 9 Biopathology and Regenerative Medicine Institute (IBIMER), Centre for Biomedical Research (CIBM), University of Granada, Granada, Spain.; 10 Department of Human Anatomy and Embryology, Faculty of Medicine, University of Granada, Granada, Spain.; 11 Excellence Research Unit "Modeling Nature" (MNat), Centre for Biomedical Research (CIBM), University of Granada, Granada, Spain.

**Keywords:** triple-negative breast cancer, drug repurposing, organoids, NDRG1, cancer stem cells

## Abstract

There is an urgent need for new therapeutic strategies against aggressive triple-negative breast cancer (TNBC), and drug repurposing offers a promising, time- and cost-effective solution. We previously reported that TGFβ leads to the tumorigenic role of NDRG1 in TNBC. Here, we aimed to identify drugs that mimic the transcriptomic signature after the inhibition of TGFβ-induced NDRG1 and to determine their antitumor properties. The transcriptomic signature was obtained by RNA sequencing after gene silencing of TGFβ-induced *NDRG1* expression in TNBC cells. For the drug repositioning study, the transcriptome was further computationally analyzed by using the Connectivity Map tool. Efavirenz, ouabain, and vinburnine were selected as the repurposed drug candidates to evaluate their therapeutic potential in TNBC models as monotherapies and pairwise combinations. We determined that the candidate drugs significantly reduced tumor cell proliferation, cancer stem cells, self-renewal, clonogenic properties, and migration abilities in TNBC cell lines through the blockade of AKT. Importantly, we validated their translational potential in TNBC patient-derived xenograft organoids in combination with docetaxel. After validating that the drugs decreased p-AKT and Ki67, we demonstrated their antitumor activity in combination with docetaxel in organoids. In addition, drugs also showed efficacy in a docetaxel-resistant TNBC model, supporting their potential to overcome chemoresistance. In conclusion, these findings demonstrate the potential of efavirenz, ouabain, and vinburnine as repurposed agents capable of inhibiting TNBC cell proliferation, stemness, and migration. Their synergistic effects with docetaxel in organoid cultures further underscore their translational relevance and highlight a promising strategy for combination therapies to improve TNBC treatment.

## Introduction

Triple-negative breast cancer (TNBC) is the most aggressive subtype, marked by the absence of estrogen (ER), progesterone (PR), and HER2 receptors, comprising 15-20% of breast cancer cases. It exhibits high relapse rates, lower survival, and poorer outcomes than other subtypes. TNBC is enriched in cancer stem cells (CSCs), a subset of cells endowed with stem-like properties, which are implicated in the sustenance of tumor growth, progression, recurrence, and metastasis, and associated with dysregulation of epithelial-mesenchymal transition (EMT)^1^. Notably, chemotherapy has remained the primary mode of treatment for TNBC, despite the availability of alternative approaches for certain cases of metastatic TNBC^2^,^3^. Numerous clinical trials are dedicated to targeting CSCs (e.g., NCT04461600, NCT02299635, NCT01151449); however, there persists an urgent need for identifying new targets and developing more specific therapies^4^. Pursuing this line of investigation and seeking novel strategies or targets related to CSCs, our previous study unraveled the pleiotropic role of NDRG1 (N-myc downstream-regulated gene 1) in TNBC. We demonstrated that exposure of TNBC cells to TGFβ1 resulted in malignant hyperphosphorylated NDRG1, which played a crucial role in inducing EMT and maintaining CSCs. Furthermore, *NDRG1* inhibition was also correlated with a reduced migration index, decreased CSC populations, and inhibition of metastasis-related genes in two different TNBC cell lines, providing a novel strategy against TNBC^5^. In addition to these findings, NDRG1 also functions as a stress-responsive protein that modulates the unfolded protein response (UPR) through the PERK/eIF2α pathway^6^. Additionally, it has been shown to stabilize the DNA repair enzyme O6-methylguanine-DNA methyltransferase (MGMT), linking NDRG1 to chemoresistance mechanisms in hypoxic tumors^7^. These diverse and context-dependent functions further support the relevance of NDRG1 as a potential therapeutic target in aggressive cancers such as TNBC.

Drug repurposing (drug repositioning or drug reprofiling) offers a strategy for identifying new applications for existing medications. Because drug candidates are already approved by the FDA and/or other regulatory agencies, and their characteristics have been extensively studied, the risks, time, and costs associated with drug development can be reduced^8^. Among various *in silico* strategies^9^, we emphasize the computational method based on transcriptional signatures, particularly the Connectivity Map (CMap) Query tool developed at the Broad Institute^10^, which has facilitated successful repurposing for various types of cancers (e.g., head and neck squamous cell carcinoma^11^ and multiple myeloma^12^). Nonetheless, these computational drug candidates necessitate *in vitro* verification to confirm the desired effects^9^.

A multitude of preclinical models to recapitulate the heterogeneity of human cancers are available. For instance, patient-derived xenografts (PDXs) are generated from a tumor biopsy implanted into immune-deficient mice and represent a highly faithful model^13^. However, these models are cost and time-consuming approaches for validating the effects of multiple drug candidates^14^. PDX organoids (PDxOs) are three-dimensional (3D) models that maintain complexity and fidelity from the tumor of origin^15^. Therefore, the establishment of PDxOs constitutes a viable, economical, and time-efficient alternative for drug screening, as demonstrated in different tumor types, such as breast^15^ or prostate cancers^16^.

In the current study, we hypothesized that drugs with transcriptomic signatures analogous to *NDRG1* knockdown under TGFβ stimulation could exert anti-tumor effects in TNBC. We aimed to identify FDA-approved drugs via the CMap online tool and assess their potential as novel therapies against TNBC by evaluating their impact on CSC properties, EMT markers, and PDxOs of TNBC.

## Materials and Methods

### Cell culture

Human TNBC cell lines MDA-MB-231 and BT549, obtained from ATCC, and SUM159 from Asterand, were cultured in Dulbecco's Modified Eagle Medium (DMEM; Sigma-Aldrich; Darmstadt, Germany) supplemented with 10% fetal bovine serum (FBS; Sigma-Aldrich) at 37°C and 5% CO_2_.

### Gene knockdown, TGFβ stimulation, and RNA sequencing

In the MDA-MB-231 cell line, *NDRG1* gene was inhibited by transient transfection with siRNA (siNDRG1) from Santa Cruz (Dallas, TX, USA) (50 ng/mL) and lipofectamine RNAiMAX (Invitrogen; Waltham, MA, USA) following the manufacturer's instructions after stimulation with TGFβ1 (10 ng/mL) (PeproTech; Waltham, MA, USA) for 8 h. Scrambled siRNA (SCR) was used as the negative control^5^.

RNA was extracted using the Illumina Ribo-Zero Plus rRNA Depletion Kit (Illumina, San Diego, CA, USA) following the manufacturer´s instructions. RNA sequencing (RNA-Seq) was conducted using a poly-A selection protocol. The final library (adapter and index included) was validated using a specific DNA chip (Agilent 2100 Bioanalyzer DNA 1000 kit). The final product was a band of approximately 265-300 bp. The sequencing was done using the NextSeq 500 platform (Illumina). Raw FASTQ files were mapped using RNA-Seq by Expectation Maximization (RSEM)^17^. Data were normalized to ensure comparability between the different samples by the TMM method, implemented in the NOISeq package^18^. Differential expression was calculated using DESeq2^19^. Genes displaying a log_2_ fold change ≥ 1.5 and FDR ≤ 0.05 were considered significant.

### CLUE Query (Clue.io from the Broad Institute)

Drug repurposing was performed using the Connectivity Map database and CMap Query tool (https://clue.io/) developed at the Broad Institute. Briefly, this tool proposes compounds characterized by a score (*tau*) from -100 to 100 that results from a comparison between the query gene signature and the transcriptional effect of thousands of *perturbagens* upon different cell lines of its database L1000^10^, where a positive score close to 100 indicates that the compound is meant to mimic the introduced gene signature. This database incorporates eight cancer-derived cell lines (only one breast cancer cell line, MCF-7) and one non-cancerous line. Additionally, each computed query returns a heat map of *perturbagen classes* (PCL), a group of compounds sharing the same mechanism of action. Results can be studied in each cell line that constitutes the database, as well as two categories that summarize the connectivity scores observed in the nine cell lines: *General* analysis' connectivity score is based on the median of *tau* scores for each cell line, whereas *Summary*'s is obtained using a maximum quantile statistic; the latter being more sensitive to signal in a subset of cell lines than the median^10^. Particularly, the differential expression of *NDRG1* knockdown, TGFβ1-treated MDA-MB-231 TNBC cell line (Table S1), which we had previously obtained by RNA-Seq (Table S1), was used to search for compounds with similar transcriptional signatures. Two queries for genes over ±1.5 and ±1.6 log_2_ fold change were performed. Compounds with* tau* higher than 80, 90, and 96 for *General, Summary,* and MCF-7 cell line analyses were considered.

### Treatments

Cells were treated with Efavirenz (E), Ouabain (O), Vinburnine (V), and Docetaxel (D) (MedChemExpress; Monmouth Junction, NJ, USA) at the indicated concentrations for cell viability assays, dissolved in DMSO (Sigma-Aldrich). The calculated half maximal inhibitory concentration (IC50) values were utilized as treatment doses for subsequent experiments. Control cells were treated with 1% DMSO as the vehicle.

### Docetaxel-resistant cell line culture

We assessed the effectiveness of the candidate drugs on a docetaxel-resistant cell line (SUM159-R), as we previously reported^20^. Briefly, the parental SUM159 line underwent 10 cycles of 48-h treatment with docetaxel (D) (10 nM) followed by 48 h of recovery without treatment. Then, 4-6 additional treatment cycles with D (50 nM) were performed.

### Cell viability assay

Cell viability and IC50 values were assessed using WST-1 reagent (Roche; Vienna, Austria). Cells were seeded in 96-well plates and treated at increasing concentrations of each compound for 72 h. WST-1 reagent was added and incubated at 37°C for 1-2 h, following the manufacturer's recommendations. The absorbance at 450 nm was measured using an M200 Nanoquant plate reader (Tecan). Cell viability is expressed as normalized absorbance (%) relative to control cells.

To study whether repurposed drugs could restore chemosensitivity to docetaxel (D), we calculated the IC50 value of D in combination with vehicle or a repurposed drug candidate for 72 h. Briefly, SUM159-R cells were seeded in 96-well plates and treated with the vehicle or the IC50 of each drug and increasing concentrations of D (nM). After 72 h, WST-1 reagent was added and incubated at 37°C for 1-2 h, following the manufacturer's recommendations. The absorbance at 450 nm was measured using an M200 Nanoquant plate reader (Tecan). Cell viability was expressed as normalized absorbance relative to control cells.

### Flow cytometry: Apoptosis, ALDH, CD44/CD24, Side Population

Cell apoptosis was analyzed with an Annexin V Apoptosis Detection Kit with propidium iodide (PI) (Immunostep; Salamanca, Spain) according to the manufacturer's protocol. Aldehyde dehydrogenase 1 (ALDH1) enzyme activity was evaluated with the Aldefluor assay kit (StemCell Technologies; Vancouver, BC, Canada) using Aldefluor buffer supplemented with Verapamil (24 μg/mL). After 72 h of treatment, cells were incubated with the Aldefluor reagent for 40 min at 37°C or DEAB (diethylaminobenzaldehyde) as the negative control. CD44^high^/CD24^-^ cell populations were assayed by staining with anti-CD44-PE and anti-CD24-FITC (BD Biosciences; San Jose, CA, USA). Briefly, 72 h after treatments, cells were collected, resuspended in PBS with 2% FBS and 1% HEPES, and incubated with anti-CD44 and anti-CD24 antibodies at 4°C for 15 min. Data acquisition was performed with a flow cytometer (FACS Verse, BD Bioscience), and their analysis was made with the FlowJo software.

The side population was determined by Hoechst 33342 dye exclusion assay. Briefly, after treatments for 72 h, cells were collected, resuspended in DMEM supplemented with 2% FBS and 10 mM HEPES, and incubated with 5 µg/mL Hoechst 33342 (Sigma-Aldrich) for 90 min at 37°C in the dark with interval mixing. Verapamil (50 µM) was used as a control of inhibition. Hoechst 33342 was excited with a UV laser at 355 nm, and emissions were detected at 450/50 nm (Hoechst blue) and 670/30 nm (Hoechst red) in a FACSAria III cell sorter (BD Biosciences). Analysis was carried out using the FlowJo software.

### Mammosphere-forming efficiency (MSFE) assay

To evaluate the self-renewal capacity of treated cells, a mammosphere-forming efficiency (MSFE) assay was performed as we previously reported^5^. Briefly, cells were cultured in 24-well ultra-low attachment plates to form primary mammospheres and treated with the corresponding treatments in DMEM/F-12 nutrient mixture without FBS, supplemented with 1X B-27 (B-27™ Supplement (50X) Minus Vitamin A; Invitrogen), 4 ng/mL heparin (Sigma-Aldrich), 10 µg/mL insulin (Insulin-Transferrin-Selenium[ITS-G 100X]; Invitrogen), 1 µg/mL hydrocortisone (Sigma-Aldrich), 10 ng/mL of epidermal growth factor (EGF, Sigma-Aldrich), 10 ng/mL fibroblast growth factor (Sigma-Aldrich), 10 ng/mL interleukin 6 (Miltenyi, Bergisch Gladbach, Germany) and 10 ng/mL hepatocellular growth factor (Miltenyi), based in patented cell culture protocol WO2016020572A1. After 72 h (SUM159, SUM159-R) or 5 days (MDA-MB-231 and BT549), primary mammospheres were manually counted, whose diameter was larger than 50µm. Then, they were collected, trypsinized, and replated without additional treatment (500 cells/well for MDA-MB-231 and SUM159-R, and 250 cells/well for BT549 and SUM159). Secondary mammospheres were counted after 3 (SUM159), 5 (BT549, SUM15-R), or 7 days (MDA-MB-231). MSFE (%) was calculated by dividing the number of mammospheres by the number of cells seeded.

### Soft agar colony assay

To determine the clonogenic capacity of the cells, we performed a soft agar colony assay as published with modifications^5^,^20^. Attached TNBC cells pretreated for 72 h were harvested and seeded at a density of 10,000 live cells in 0.4% agar DMEM on top of a 0.8% agar DMEM layer in 24-well culture plates. The surface was covered with cell media without additional treatments. Cell media was replaced every 4 days, and the cells were incubated for 21 (SUM159) and 28 days (MDA-MB-231). Colony formation (diameter >50 µm) was examined under a microscope after staining with Iodonitrotetrazolium chloride (Sigma-Aldrich). For colony counting, images of the dishes were captured with a Leica Si9 magnifying glass and analyzed with ImageJ. The images were converted to 8-bit format, and the threshold was adjusted to detect the colonies. Colonies with a diameter greater than 50 μm were then counted using the “analyze particles” function.

### Wound-healing assay

Cells were seeded in 6-well plates until 80% confluence and were treated for 24 h. At this confluence, no changes in cell proliferation were observed. Then, a wound was made in the cell monolayer with a 100 µl-pipette tip. After washing with PBS, fresh medium was added. Photos were taken at 0, 14 h (MDA-MB-231, BT549), and 24 h (SUM159) after the wound was made with an Olympus CKX53 bright-field microscope. Cell migration was analyzed with ImageJ, as we have previously published^5^.

### qPCR

Following the manufacturer's instructions, RNA was extracted using GeneJET RNA Purification Kit (Thermo Fisher Scientific; Waltham, MA, USA). cDNA was synthesized using the iScript cDNA Synthesis Kit (Bio-Rad, Hercules, CA, USA) following the manufacturer's instructions. Quantitative PCR (qPCR) was performed using iTaq Universal SYBR Green Supermix (Bio-Rad) in a QuantStudio 6 Real-Time PCR System (Thermo Fisher Scientific) for single-tube assays. Sequences of gene-specific primers, spanning exon-exon junctions in the target mRNA, used for single-tube qPCR are available in Table S2. The following thermal conditions were used: a single cycle of 95°C for 10 min, followed by 40 cycles of 95°C for 15 s and 60°C for 60 s. The mRNA abundance was normalized based on GAPDH content. The determination of 18S rRNA was used as a quality control check among samples. Detection of amplified products was carried out using SYBR Green I. Expression levels were calculated as 2^-∆∆Ct^, where ∆Ct was the difference of Ct in each sample between the target gene and the normalizer, as we published^20^.

### Western blot

Cells were collected and lysed in 1X lysis buffer (Cell Signaling; Danvers, MA, USA) and 1X protease/phosphatase inhibitor cocktail (Thermo Fisher Scientific). Samples (30 μg protein) were boiled in sample buffer (Thermo Fisher Scientific) containing β-mercaptoethanol (Sigma-Aldrich), separated by SDS-PAGE electrophoresis in polyacrylamide gels, and transferred onto nitrocellulose membranes (Bio-Rad; Hercules, CA, USA), as we previously published^5^,^20^. Membranes were incubated overnight at 4°C with the primary antibody (1:1000 dilution) and the secondary antibody for 1 h (1:2000 dilution). Antibodies against AKT from BD Biosciences; p-AKT (Ser473), Snail (C15D3), Slug (C19G7), Vimentin (D21H3), p-NDRG1 (Thr346, D98G11), and NDRG1 (D8G9) were from Cell Signaling; GAPDH (1E6D9) was from Proteintech (Waltham, MA, USA). Membranes were incubated with chemiluminescence reagents to detect the protein expression and visualized using the ImageQuant LAS4000. Densitometric analysis was performed using ImageJ.

### Patient samples

The study recruited one patient diagnosed with TNBC from the Oncology CMU of the Hospital Universitario Clinico San Cecilio (Granada, Spain). After signing the patient´s informed consent, a core needle tumor biopsy was collected according to the protocol approved by the Reference Ethics Committee with code PI19/01533/1626-N-19.

### Tumor tissue and patient-derived xenografts (PDXs)

We generated a patient-derived xenograft (PDX) (UGR01) model from a core needle biopsy of a TNBC patient enrolled at the Hospital Universitario Clinico San Cecilio (Granada, Spain), as we published^21^. Briefly, tumor biopsy (1 mm³) was orthotopically implanted into the cleared mammary fat pad of 4- to 8-week-old female NOD SCID gamma mice (NSG, *NOD. Cg-Prkdcscid Il2rgtm1Wjl/SzJ*). PDX tumor tissue (G0) was excised and cut into small (1 mm³) fragments and then re-implanted in new mice (4 mice) to obtain G1. This process was repeated until G2 was generated. When G2 tumors reached 120-170 mm³ in size, mice were sacrificed, and the tumors were collected. All animals were housed and maintained at 20-24°C, 50% relative humidity, and a 10:14h light-dark cycle with food and water *ad libitum.* Early generations of the PDX tumor tissue were fixed in 4% paraformaldehyde (PFA) and embedded in paraffin for further histopathological characterization by a pathologist.

### Establishment of patient-derived xenograft organoid (PDxOs) cultures

For the establishment of the PDxOs cultures, PDX tumor tissue was finely minced and digested enzymatically with collagenase A (1.6 U/mL) (Roche) for 15 min at 37°C in an orbital shaker (200 rpm/min). After precipitation, the supernatant was collected and preserved on ice. Two or three additional digestions were performed to enrich the pellet. Then, red blood cells were eliminated by using an ammonium chloride solution. Isolated cells were embedded in growth factor-reduced solubilized basement membrane (Matrigel^®^, Corning; Glendale, AZ, USA) or Basement Membrane Extract type 2 (R&D Systems; Minneapolis, MN, USA) and plated into 50 µl drops in 24-well plates. After 30-60 min at 37°C, the organoid medium was added and changed every 3-4 days. Organoid medium consists of Advanced DMEM/F12 with 5% FBS, 10 mM HEPES, 1X Glutamax, 1X Penicillin/Streptomycin, 10 ng/mL hEGF, and 10 μM Y-27632^15^,^22^. When the PDxOs were mature and confluent, they were passaged, usually once a week, with Cell Recovery solution (Corning) for 1 h at 4°C. Later, PDxOs were trypsinized to single cells at 37°C, and after neutralization and a wash with PBS, cells were re-embedded as described above.

### Immunohistochemistry and FISH of PDX and PDxOs

When PDxOs reached 100 µm in diameter, they were collected, incubated with Cell Recovery solution (Corning) for 1 h at 4°C, fixed in 4% PFA for 10 min at RT, and embedded in Agarose 1%. PDX tumor tissue was fixed in 4% PFA at 4°C for 24 h. Then, PDxOs and PDX were washed in 0.1 M PBS, embedded in paraffin with an automatic tissue processor (TP1020; Leica, Germany), and cut into sections (4 µm). Immunohistochemistry (IHC) was performed and analyzed by a breast cancer pathologist, as we published^21^. Briefly, antigen retrieval was performed using Antigen Retrieval fluid 10X EDTA (pH 8.0, Vitro). Staining was made in an Autostainer 480 (Vitro) by using the Master Polymer Plus Detection System (Peroxidase) (Vitro). After antigen retrieval, sections were washed and blocked with 3% hydrogen peroxide for 5 min. Primary antibodies against ER (rabbit monoclonal antibody, clone SP1; Vitro) and PR (rabbit monoclonal antibody, clone 16; Vitro) were applied for 5 and 10 min at room temperature, respectively. As a negative control, the primary antibody was replaced by a non-immune serum. Sections were then treated with immunodetection solution (biotinylated secondary antibody) for 30 min, and 3,3'-diaminobenzidine (1:50 dilution) (Vitro) as the chromogenic agent. Sections were counterstained in Meyer's hematoxylin. A pathologist considered the absence of any nuclear staining in neoplastic cells as negative. Images were acquired on a Leica DM 550B microscope. HER2 status was determined using immunohistochemistry with antibodies against HER2 (vitro 1:100) for PDxOs and the fluorescence in situ hybridization (FISH) test in the deparaffinized sections of PDX tumor tissue (ERBB2/CCP17 FISH Probe Kit, CT-PAC001, CytoTest Inc). The latest ASCO/CAP 2023 recommendations for condition assessment were used for both interpretations by a pathologist^23^.

### Flow cytometry of PDxOs

The murine population of PDxOs was determined by assessing the Major Histocompatibility Complex (MHC) Class I (H-2Kd) through flow cytometry. Briefly, after the dissociation of PDxOs into single cells, they were incubated with anti-mouse MHC Class I (H-2Kd) (1.2 µg/mL, SF1-1.1.1, Invitrogen) for 15 min at room temperature (RT). Data acquisition and analysis were made using a flow cytometer (FACS Verse, BD Bioscience) and the FlowJo software, respectively.

### Cell viability in PDxOs

PDxOs were passaged and grown for three days. When their diameter was around 30-50 µm, they were harvested and re-embedded in 10 µl/well of Matrigel^®^ in a 48-well plate. Organoids were treated with vehicle (DMSO), D, E, O, or V at increasing concentrations for three days. Cell viability was assessed by adding 10% AlamarBlue HS (Invitrogen) to each well and incubation for 6h. Fluorescence (emission wavelength: 590 nm) was read using an M200 Nanoquant plate reader (Tecan). Cell viability was expressed as normalized fluorescence relative to control cells. IC50 values were calculated using GraphPad Prism 9.0.0 software.

### Immunofluorescence of PDX and PDxOs

Immunofluorescence of PDX tumor tissue was performed as we published^5^,^21^. For PDxOs, after ten days of growth in maintenance, they were plated in an 8-well chamber for NDRG1 basal detection. Nonetheless, in the case of p-AKT and Ki67 detection, after 6 days of growth, PDxOs were plated in an 8-well chamber and treated at the IC50 of each drug or vehicle for 72 h. Then, organoid samples were washed with PBS, fixed with 4% PFA for 20 min at RT, permeabilized with 0.2% Triton X-100 for 20 min at RT, and blocked with 3% BSA for 1 h at RT. PDxOs were incubated with primary antibodies against NDRG1 (D8G9, Cell Signaling) (1:100 dilution) or p-AKT (Cell Signaling) (1:100 dilution), and Ki67 (8D5, Cell Signaling) (1:800 dilution) in BSA 0.1% overnight at 4°C. Samples were washed thrice with PBS and incubated with secondary antibodies anti-rabbit Alexa Fluor 488 (Cell Signaling) (1:500 dilution) and anti-mouse Alexa Fluor 594 (Cell Signaling) (1:500 dilution) for 2 h at RT in the dark. Then, they were washed thrice with PBS, and nuclei were counterstained with Hoechst (Sigma-Aldrich) (1:100 dilution) for 15 min at RT in the dark and washed thrice with PBS to remove the excess. Finally, slides were mounted with a mounting medium (Cell Signaling). Images were taken by a confocal microscope Zeiss LSM 710, and staining intensity analyses were performed with ImageJ.

### Statistical analysis

Results are shown as mean ± standard deviation (SD). Statistical differences between two experimental groups were analyzed using a two-tailed Student's t-test with GraphPad Prism 9.0.0 (GraphPad Software Inc.). The experiments were conducted in the number of replicates specified in the figure legends. A *p*-value ≤ 0.05 was considered statistically significant.

## Results

### Selection of drug candidates based on NDRG1 knockdown in TGFβ1-stimulated TNBC cells

**Figure 1:**
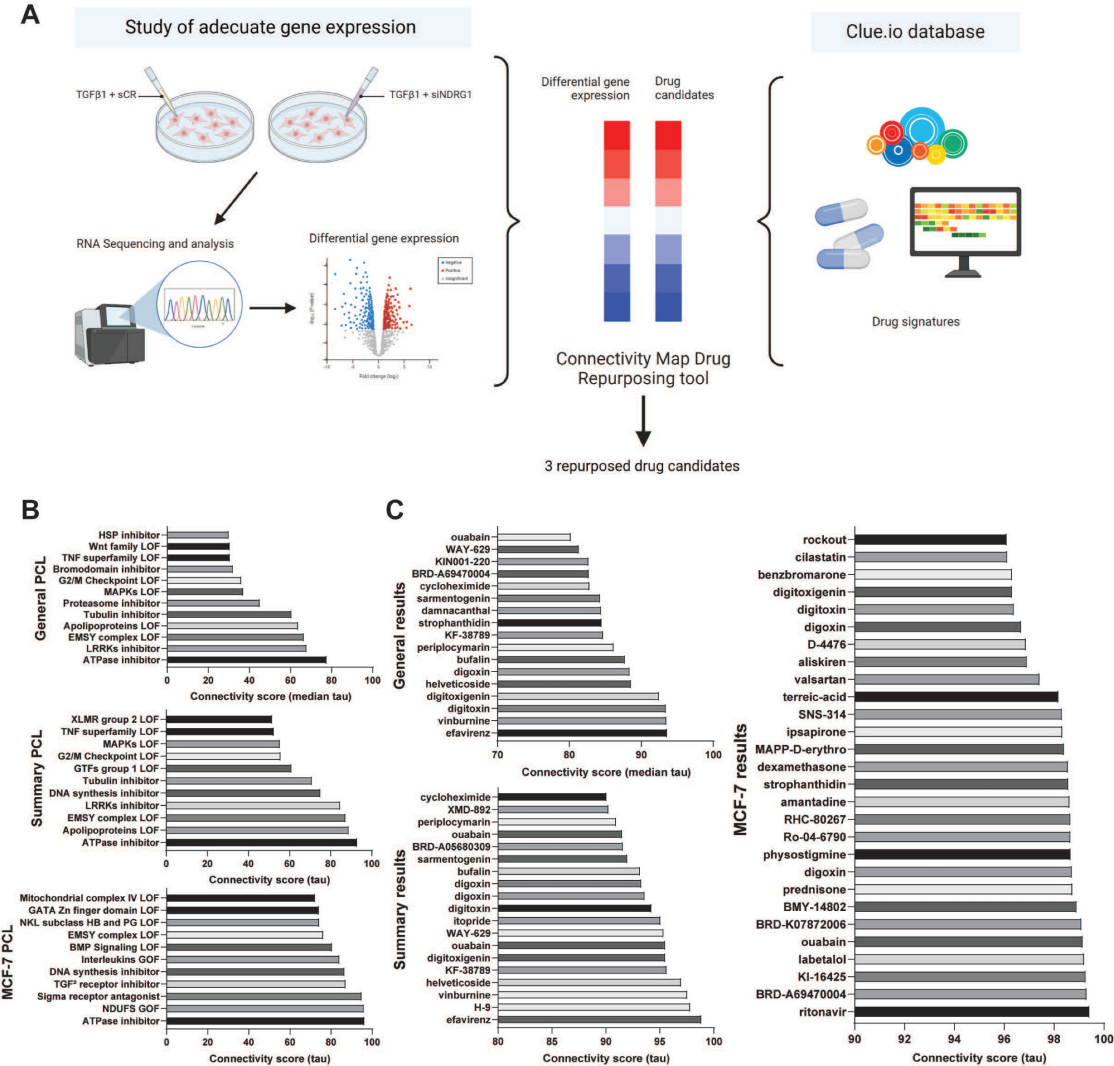
** Study and selection of repurposed drug candidates using Connectivity Map Clue.** (A) Schematic illustration of the drug repurposing process using Connectivity Map Clue. (B) Perturbagen class (PCL) topmost connectivity scores of ±1.6 log_2_ fold change query. (C) Compounds with connectivity scores higher than 80, 90, and 96 for General, Summary, and MCF-7 cell line analyses, respectively, of ±1.6 log_2_ fold change query. Bone Morphogenetic Protein (BMP); Gain Of Function (GOF); General Transcription Factors (GTFs); Heat Shock Protein (HSP); Loss Of Function (LOF); Leucine Rich Repeat Kinases (LRRKs); Mitogen-Activated Protein Kinases (MAPKs); NADH ubiquinone oxidoreductase core subunits (NDUFS); NK-Like (NKL); Transforming Growth Factor (TGF) Tumor Necrosis Factor (TNF); X-Linked Mental Retardation (XLMR).

To perform a drug repurposing study based on the gene expression signature resulting from *NDRG1* knockdown in MDA-MB-231 cells under TGFβ1 stimulation using CMap (Fig. 1A), we first conducted an RNA-Seq analysis to assess this differential signature. After data curation, based on statistical significance, we limited the expression level by selecting a log_2_ fold change over ±1.5 (Table S1) to achieve a restrictive differential gene expression. As expected, among down-regulated genes, *NDRG1* was highly inhibited (log_2_ fold change = -2.796). We also highlighted the inhibition of *CXCL10* and *CXCL11*, *PODXL,* and *OAS1.* Then, as previously detailed, using this differential signature (Table S1), we conducted two queries in the CMap tool: one with a log_2_ fold change limit set at ±1.6 (Fig. 1B, 1C), and another with a limit set at ±1.5 (Fig. S1A, S1B). We aimed to replicate the antitumor transcriptional characteristics observed upon *NDRG1* inhibition following TGFβ1 stimulation. Therefore, we focused on PCL and the compounds that exhibited positive connectivity scores. We focused our investigation on three categories of results:* General*, *Summary,* and MCF-7, as the only breast cancer cell line available in the CMap tool.

Results of PCL analysis (Fig. 1B, S1A) showed that *NDRG1* knockdown was associated with events such as mitogen-activated protein kinases (MAPKs), WNT family, and histone-modifying EMSY complex loss of function, and the expression of TGFβ receptor inhibitors. Upon comparing the results of both queries, we observed higher connectivity scores and greater similarity across different categories in the ±1.6 log_2_ fold change results compared to the ±1.5 log_2_ fold change results. Specifically, ATPase inhibitors emerged as the strongest result in all three categories of the ±1.6 log_2_ fold change query (Fig. 1B). Taken together, these findings suggested that a more restrictive gene expression threshold yields more precise results.

Later, we focused on compounds with connectivity scores higher than 80, 90, and 96 for *General*, *Summary*, and MCF-7 cell line analyses, respectively, in the ±1.6 log_2_ fold change query, as illustrated in Fig. 1C. Unsurprisingly, *General* and *Summary* analyses overlapped in most of the compounds, similar to the PCL analyses. The top two compounds in both *General* and *Summary* results were efavirenz and vinburnine. Additionally, several ATPase inhibitors, such as digitoxigenin, digitoxin, digoxin, and ouabain, appeared in both categories, consistent with their ranking as the top PCL result (Fig. 1B). Despite the broader range of drugs identified in the MCF-7 analysis, we highlighted the continued presence of these ATPase inhibitors.

Comparing the results of the ±1.6 and ±1.5 queries, we found that the ±1.6 query (Fig. 1C) yielded more drug candidates with strong connectivity scores. Consequently, we chose to proceed with the ±1.6 results for our study. Notably, several drugs with high connectivity scores in the ±1.5 query (Fig. S1B) did not appear in the ±1.6 query (Fig. 1C), such as tropisetron, ABT-751, or vincristine in the *General* category, and MK-7108, epothilone, or tropisetron in the *Summary* category.

From the drugs presented in Fig. 1C, we successfully identified several candidates, avoiding those with the same mechanism of action, and proposed three candidates as summarized in Table 1. Based on the *General* and *Summary* results, we preferred efavirenz and vinburnine, and we selected ouabain as the ATPase inhibitor with the highest connectivity score in the MCF-7 cell line.

### Repurposed drug candidates compromised cell viability and induced cell apoptosis

**Figure 2:**
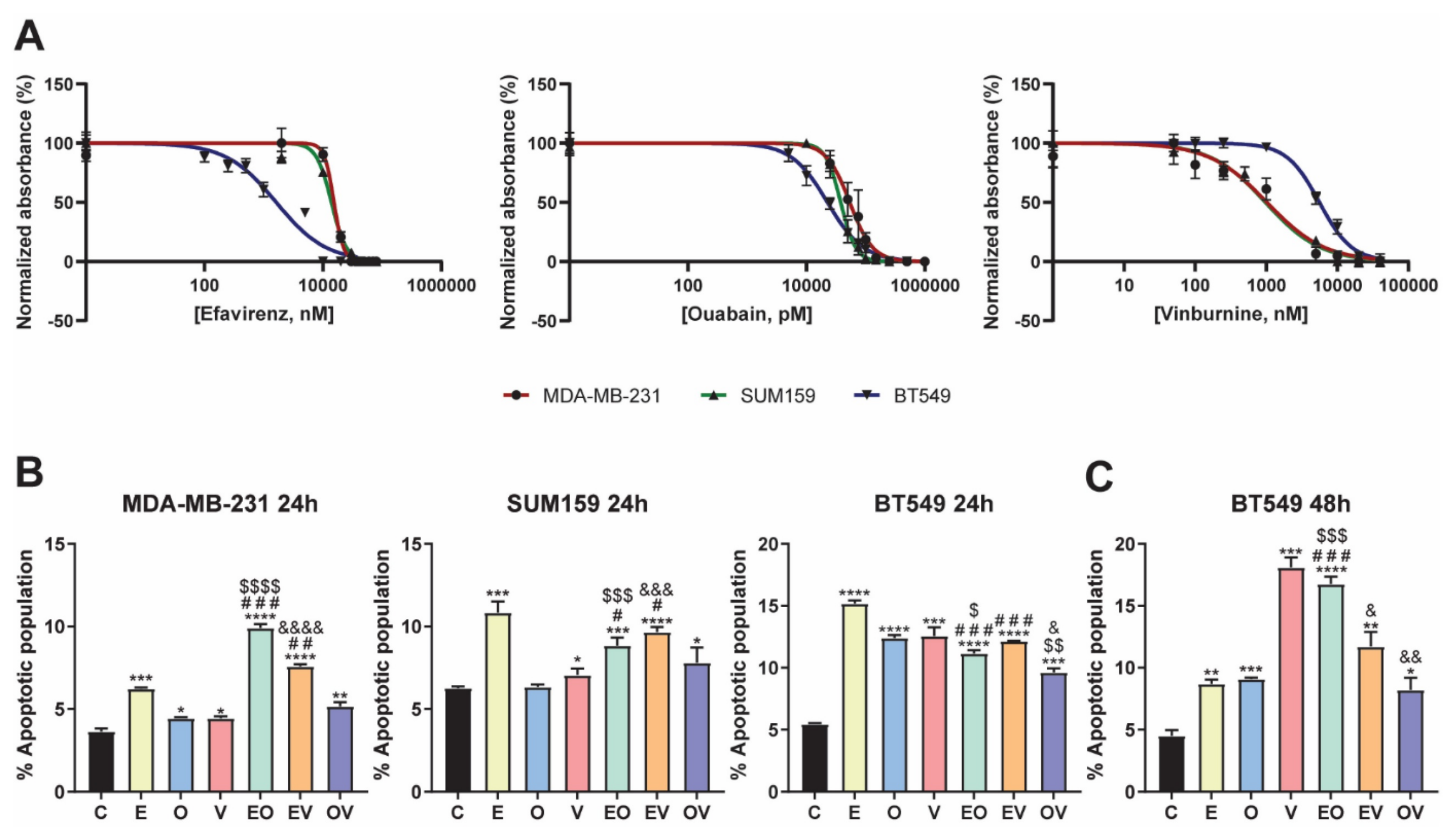
**Repurposed drug candidates compromised cell viability and induced apoptosis**. (A) IC50 of Efavirenz (E), Ouabain (O), and Vinburnine (V) in MDA-MB-231, SUM159, and BT549 cell lines after 72-h treatments. Results were normalized to the vehicle control. Data are presented as mean ± SD (n=6). (B) Flow cytometric analysis of total apoptotic population after 24-h treatments in MDA-MB-231, SUM159, and BT549 cell lines. Data are presented as mean ± SD (n=3). (C) Flow cytometric analysis of total apoptotic population after 48-h treatments in the BT549 cell line (n=3). Statistically significant differences with the vehicle (C): * P ≤ 0.05, ** P < 0.01, *** P < 0.001, **** P < 0.0001. Statistically significant differences with E: # P ≤0.05, ## P < 0.01, ### P < 0.001. Statistically significant differences with O: $ P ≤ 0.05, $$ P < 0.01, $$$ P < 0.001, $$$$ P < 0.0001. Statistically significant differences with V: & P ≤ 0.05, && P < 0.01, &&& P < 0.001, &&&& P < 0.0001. Data are presented as mean ± SD.

To determine whether our repurposed drug candidates were effective for the treatment of TNBC, we first studied their effect on the viability of three different cell lines for 72 h of treatment. Drug treatments were performed without TGFβ1 stimulation. This decision was based on the fact that the RNA-seq analysis had already compared *NDRG1* inhibition in TGFβ1-treated cells versus TGFβ1-stimulated controls, generating a gene signature that inherently reflects the effects of TGFβ1 stimulation. Consequently, the Connectivity Map identified compounds resembling this gene signature, which already accounts for the impact of TGFβ1. All cell lines were sensitive to the three repurposed drugs (Fig. 2A), and the calculated IC50 values are summarized in Table 2. In the case of efavirenz (E), MDA-MB-231 and SUM159 were similarly affected, exhibiting IC50 values of 15.4 and 13.9 µM, whereas BT549 was more sensitive with an IC50 of 1.6 µM. Regarding ouabain (O) treatment, our results showed that it presented the most toxic effect in all cell lines, achieving the lowest IC50 values among all drugs: 54.0, 38.0, and 22.7 nM, in MDA-MB-231, SUM159, and BT549, respectively. Finally, when treated with vinburnine (V), while the IC50 of MDA-MB-231 and SUM159 were again similar with a value of 1.0 µM in both cell lines, BT549 was more resistant, exhibiting a higher IC50 of 5.6 µM, in contrast to the behavior against E. We established these specific treatment doses for subsequent experiments, as shown in Table 2.

After this selection, we assessed whether our repurposed drugs induced apoptosis after 24 h of treatment. As depicted in Fig. 2B and Fig. S2A, in MDA-MB-231 and SUM159, E was the best candidate for increasing the total apoptotic population. Moreover, we aimed to investigate the effects of pairing these three drugs in combinations, namely efavirenz + ouabain (EO), efavirenz + vinburnine (EV), and ouabain + vinburnine (OV). Our findings revealed that in the MDA-MB-231 cell line, EO and EV induced higher levels of apoptosis compared to individual agents. Conversely, in SUM159, where the effect of E alone was approximately 10%, no bigger induction was observed with the combinations. On the other hand, in BT549 at 24 h, all treatments highly induced apoptosis versus control and masked the results exhibited by the combinations (Fig. 2B, Fig. S2A). Next, we investigated if this proapoptotic capability was maintained after 48 h of treatment in BT549 (Fig. 2C, Fig. S2B). As a result, we found that V increased apoptosis up to 17% and EO approximately doubled the apoptotic population compared to the two monotherapies, while EV and OV combinations maintained similar results as observed at 24 h (Fig. 2B, 2C). Overall, the cytotoxic effect was consistent with the increase observed in the apoptotic population in all cell lines (Fig. 2B, 2C).

### Repurposed drugs decreased CSC phenotypic properties and migration

**Figure 3:**
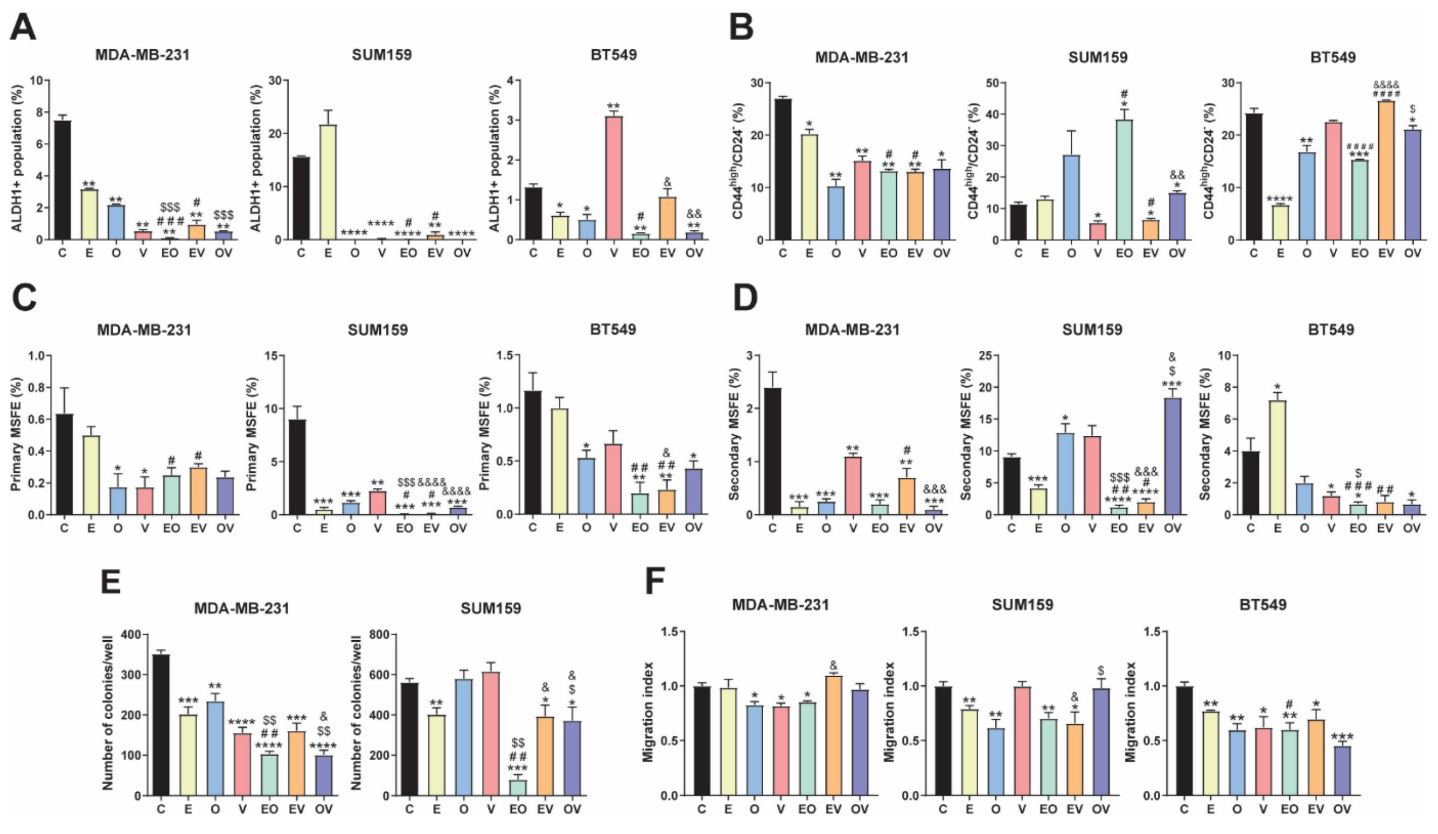
**Repurposed drugs decrease cancer stem cell (CSCs) markers and migration**. (A) Flow cytometric analysis of ALDH1+ CSC population after 72-h treatments of Efavirenz (E), Ouabain (O), Vinburnine (V), and combinations in MDA-MB-231, SUM159, and BT549 cell lines (n=2). (B) Flow cytometric analysis of CD44^high^/CD24^-^ population after 72-h treatments in MDA-MB-231, SUM159, and BT549 cell lines (n=2). (C) Mammosphere-forming efficiency (MSFE) in primary mammospheres after 72-h treatments in MDA-MB-231, SUM159 (n=4), and BT549 cell lines (n=3). (D) MSFE in secondary mammospheres in MDA-MB-231, SUM159 (n=4), and BT549 cell lines (n=3). (E) Soft-agar colony formation after 72-h treatments in MDA-MB-231 and SUM159 (n=4). (F) Tumor cell migration index after 24-h treatments in MDA-MB-231, SUM159, and BT549 cell lines (n=3). Results were normalized to the vehicle control (C). Statistically significant differences with the vehicle: * P ≤ 0.05, ** P < 0.01, *** P < 0.001, **** P < 0.0001. Statistically significant differences with E: # P ≤ 0.05, ## P < 0.01, ### P < 0.001, #### P < 0.0001. Statistically significant differences with O: $ P ≤ 0.05, $$ P < 0.01, $$$ P < 0.001. Statistically significant differences with V: & P ≤ 0.05, && P < 0.01, &&& P < 0.001, &&&& P < 0.0001. Data are presented as mean ± SD.

We assessed different functional assays to study the effect of repurposed drugs and combinations. Firstly, we observed the ALDH1+ population by flow cytometry at 72 h, as illustrated in Fig. 3A and S3A. In MDA-MB-231, all treatments produced an outstanding decrease in the ALDH1+ population. In SUM159, similar results were obtained except for E, which was not significant *versus* the control group. However, in BT549, V enhanced the ALDH1+ population, and no differences were assessed in EV, whereas with E, O, EO, and OV treatments, it decreased to almost disappear, like in the other two cell lines. We highlighted that EO and OV were the best combinations for all cell lines.

Next, we studied the CD44^high^/CD24^-^ population^24^. In contrast to the ALDH1+ population, we found different behaviors between treatments and cell lines. In MDA-MB-231, all treatment groups presented statistically significant decreases in the CD44^high^/CD24^-^ population. Nonetheless, in SUM59, V and EV reduced this population, whereas EO and OV produced significant increments. In BT549, V did not induce changes in the population, but E, O, and their combination decreased it (Fig. 3B and S3B).

We also investigated the effects of treatments in the side population, in MDA-MB-231 and SUM159 cell lines (Fig. S4). We observed a decrease in all treatments except in MDA-MB-231 with OV and EV. Altogether, this data suggested that repurposed drugs are effective in decreasing different CSC populations, especially in MDA-MB-231.

Self-renewal and colony formation capacity were evaluated by the mammosphere-forming efficiency (MSFE) and the clonogenic capacity assays, respectively. As depicted in Fig. 3C and S5A, a tendency for a decrease in primary MSFE was seen in all cell lines, especially in SUM159. However, in the second generation of mammospheres, we found in MDA-MB-231 that all treatments were highly effective in decreasing MSFE, whereas in SUM159, only E and its combinations reduced it. In BT549 cells, V, EO, and OV presented statistically different decreases versus vehicle (Fig. 3D, S5B). In summary, EO was the best candidate among all cell lines, even though E and O single agents were less effective separately. Similarly, we conducted a soft agar colony assay to evaluate the impact of our repurposed drugs on clonogenicity. Despite culturing all cell lines, BT549 did not form proper colonies. In MDA-MB-231, all treatments significantly reduced colony formation, achieving the best results in the EO and OV groups (Fig. 3E, S6A). In the SUM159 cell line, O and V did not induce changes, while E, EV, and OV caused a slight decrease, and EO emerged as the most effective treatment (p< 0.001).

Finally, cell migration was assessed using a wound-healing assay, as shown in Fig. 3F and S6B. Surprisingly, lower effects were observed in MDA-MB-231, where only treatments with O, V, and EO significantly decreased the migration index. In contrast, in the SUM159 cell line, E, O, EO, and EV led to a notable reduction in migration, whereas in BT549, all therapies significantly reduced the migration index. As previously summarized in the evaluation of CSC markers, EO appeared to be the most potent combination, yielding the best results across all cell lines and assays.

### Repurposed drugs inhibit p-AKT and EMT markers

Even though our CMap reposition was based on a transcriptional signature and our drugs did not aim to inhibit NDRG1, we evaluated by western blot the expression of both total NDRG1 and its phosphorylated form at Thr346 (p-NDRG1) at 24 and 72 h of treatment with and without stimulation with TGFβ1 (Fig. S7). As expected, we did not find NDRG1 inhibition after treatments in none of the cell lines.

**Figure 4:**
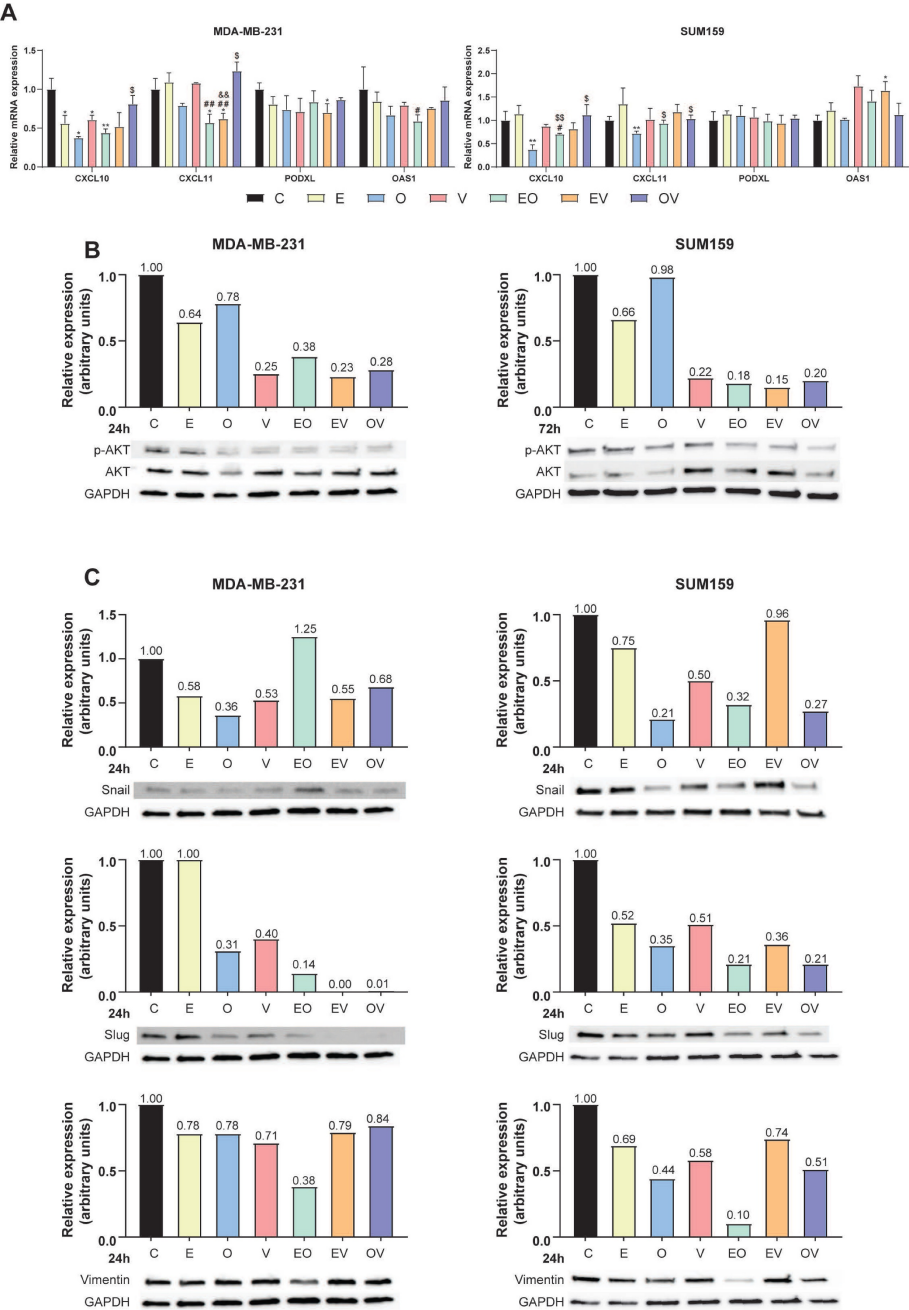
**Repurposed drugs inhibit the AKT pathway and EMT markers.** (A) Relative mRNA expression of *CXCL10*, *CXCL11*, *PODXL,* and *OAS1* after 12-h treatments of Efavirenz (E), Ouabain (O), Vinburnine (V), and combinations in MDA-MB-231 and SUM159 (n=3). (B) Western blot of p-AKT (Ser473) and total AKT and densitometric quantifications of p-AKT relative to total AKT and GAPDH levels after 24-h treatments in MDA-MB-231 and after 72-h SUM159 cell lines. (C) Western blot of EMT markers (Snail, Slug, and Vimentin) and densitometric quantifications relative to GAPDH levels after 24-h treatments in MDA-MB-231 and SUM159 cell lines. Results were normalized to the vehicle control (C). Statistically significant differences with the vehicle: * P ≤ 0.05, ** P < 0.01. Statistically significant differences with E: # P ≤ 0.05, ## P < 0.01. Statistically significant differences with O: $ P ≤ 0.05, $$ P < 0.01. Statistically significant differences with V: && P < 0.01. Data are presented as mean ± SD.

To gain insight into the antitumor mechanism of the candidate molecules and considering that this method of drug repurposing predicts molecules that are capable of imitating the transcriptional signature, we studied four genes whose expression was decreased in the original query by qPCR (Table S1), namely *CXCL10*, *CXCL11*, *PODXL,* and *OAS1*. The selection of these genes was based on their relationship with tumorigenic processes such as the AKT pathway and EMT^25^-^29^. As shown in Fig. 4A, *CXCL10* significantly decreased with monotherapies E, O, and V (* P ≤ 0.05) and the combination EO (** P < 0.01) in MDA-MB-231. However, this reduction was only statistically significant with O (** P < 0.01) in SUM159. On the other hand, *CXCL11* expression was decreased after the treatment with EO and EV in MDA-MB-231 (* P ≤ 0.05) and O in SUM159 cells (** P < 0.01). Regarding *PODXL* expression, we found a significant reduction in MDA-MB-231 cells treated with EV (* P ≤ 0.05). Lastly, *OAS1* expression was significantly enhanced in SUM159 cells treated with EV (* P ≤ 0.05). Briefly summarized, qPCR results revealed more pronounced changes in the MDA-MB-231 cell line and *CXCL10* mRNA expression, suggesting a possible relevance of the cell line origin data set and validating the efficacy of drug repurposing in targeting specific genes in TNBC.

It is known that CXCL10 and CXCL11 activate the AKT pathway via CXCR3^27^, while PODXL and OAS1 are involved in EMT through AKT modulation^26^,^29^. Based on this, we hypothesized that our compounds may exert anticancer effects via AKT. Accordingly, we assessed the expression of total and phosphorylated (Ser473) AKT (p-AKT) by western blot in MDA-MB-231 and SUM159 cells treated for 24 and 72 h (Fig. 4B, Fig. S7B). While all treatments demonstrated an overall trend toward reduced p-AKT levels, the temporal progression of these effects exhibited cell line-specific patterns. Our results showed that in MDA-MB-231 cells, pharmacological interventions induced maximal suppression of p-AKT at 24 h post-treatment (Fig. 4B), with subsequent restoration to baseline levels by 72 h (Fig. S7B). Conversely, SUM159 cells displayed distinct kinetics: V treatment transiently elevated p-AKT expression at 24 h (Fig. S7B), while this effect was absent at 72 h (Fig. 4B). The rest of the treatments induced a decrease in the phosphorylated form at 24 h (Fig. S7B) that persisted through the 72-h observation period, except for O (Fig. 4). Then, we analyzed the expression of EMT markers (Snail, Slug, and Vimentin). As illustrated in Fig. 4C, after 24 h of treatment, all treatments reduced Slug expression in both cell lines except E in MDA-MB-231. However, Snail expression was slightly different in the two cell lines. While in MDA-MB-231 all treatments produced a reduction except for EO, in SUM159, EV was the combination that did not change the expression of Snail. The rest of the treatment groups decreased the protein expression. Regarding Vimentin, we found that all the treatments slightly reduced its expression in both cell lines in MDA-MB-231, except for EO, which especially decreased it. In the SUM159 cell line, the reduction in Vimentin expression was more pronounced, especially after O and EO treatments.

### Repurposed drugs are effective in patient-derived xenograft organoids of TNBC

**Figure 5:**
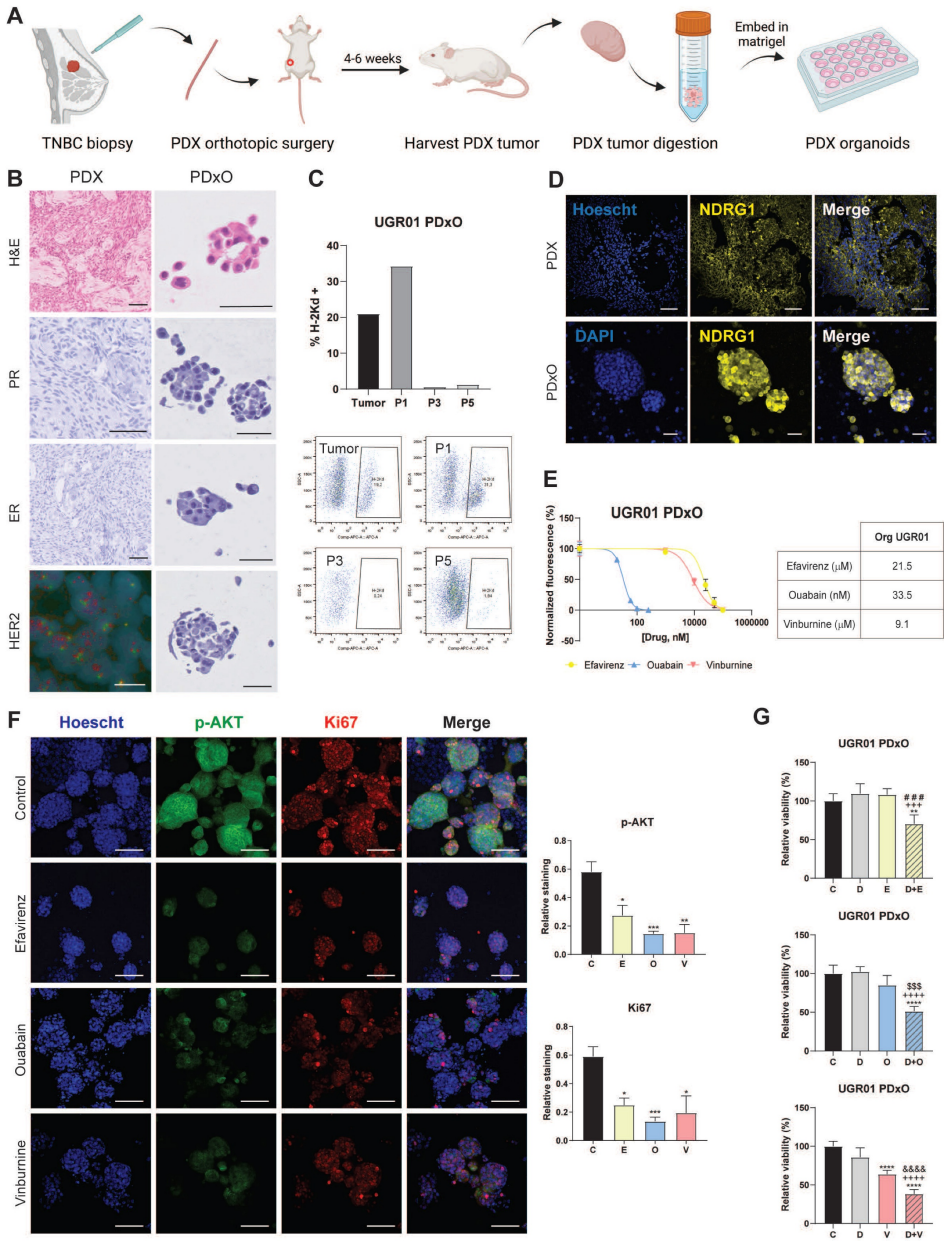
**Repurposed drugs are effective in patient-derived xenograft organoids (PDxOs) of TNBC.** (A) Schematic illustration of the establishment of PDxOs (UGR01). (B) Hematoxylin and eosin (H&E) staining, immunohistochemistry of PR (progesterone receptor) and ER (estrogen receptor) of UGR01 PDX and PDxOs, assessment of negative HER2 amplification by immunohistochemistry of UGR01 PDxOs and by FISH showing two copies of the gene (red) and centromere 17 (green) per nucleus (blue) of UGR01 PDX. The scale bar of immunohistochemistry images is 50 µm. Scale bar of FISH image 10 µm. (C) Flow cytometric analysis and plots of the H-2Kd positive staining population of UGR01 PDxOs after tumor digestion and passages (P) 1, 3, and 5 (n=1). (D) Representative confocal images of NDRG1 (yellow) in the PDX model and PDxOs (UGR01). Scale bar 50 µm. (E) IC50 of Efavirenz (E), Ouabain (O), Vinburnine (V) in the UGR01 PDxOs model after 72-h treatments. Results were normalized to the vehicle control (n=5). (F) Representative confocal images (original optical objective: 10X) and quantification of p-AKT (green) and Ki67 (red) in UGR01 PDxOs after 72-h treatments. Scale bar 100 µm (n=4). (G) Relative viability of UGR01 PDxOs after 72-h IC50 treatments of vehicle control (C), docetaxel (D), E, O, V, or combinations (D+E, D+O, D+V) (n=5). Results were relativized to the vehicle control. Statistically significant differences with the vehicle: * P ≤ 0.05, ** P < 0.01, *** P < 0.001, **** P < 0.0001. Statistically significant differences with docetaxel: +++ P < 0.001, ++++ P < 0.0001. Statistically significant differences with E: ### P < 0.001. Statistically significant differences with O: $$$ P < 0.001. Statistically significant differences with V: &&&& P < 0.0001. Data are presented as mean ± SD.

We validated the translational potential of the repurposed candidates as future co-adjuvants of conventional cancer therapies in PDxO cultures of our TNBC PDX model UGR01. We established the PDxO cultures (Fig. 5A) and further characterized that PDX and PDxOs recapitulated the histological features of the patient of origin by assessing hormone receptors (ER and PR) and HER2. Our results of IHC and FISH confirmed that both tumor and organoids maintained the TNBC subtype (Fig. 5B). Additionally, our data revealed a reduction of the murine counterpart to irrelevance from passages 3-5 (Fig. 5C), as we assessed the presence of murine population in the PDxO cultures by flow cytometry of the murine major histocompatibility complex (H2kD). Confocal microscopy confirmed the basal expression of NDRG1 in the PDX and its maintenance in the PDxO model (Fig. 5D). In summary, UGR01 is a suitable model for our drug repurposing study to validate the translational potential of our drug candidates.

As shown in Fig. 5E, similar IC50 values were obtained for O (33.5 nM) compared to the TNBC-attached cell lines. On the contrary, slightly superior values for E and V (21.5 µM and 9.1 µM, respectively) in the PDxOs (Fig. 5E). Additionally, we confirmed the antitumor potential of E, O, and V by the substantial reduction of Ki67 by IF staining (Fig. 5F), a classical proliferation marker^30^. Furthermore, our data from confocal microscopy indicated a marked reduction in p-AKT by all three monotherapies compared to control (Fig. 5F, S8A) after 72 h of treatment. This decrease in p-AKT staining corroborates the mechanism pathway of the drugs that we observed previously (Fig. 4B).

Finally, we did not test the combinations of the drugs repurposed in the present study as they have not yet received FDA approval, and further testing would extend the timeline for clinical application. However, to expedite the translation of these findings into clinical practice, we proposed an approach similar to the one observed in clinical trials, where new drugs are combined with a standard treatment^31^. Particularly, we proposed to study the combination of E, O, and V with docetaxel (D), which is a taxane that remains the standard chemotherapy of care for unresectable TNBC^4^. We found that the IC50 of D in the PDxO cultures was 8.8 nM (Fig. S8B). Further, to investigate whether the combination of D and repurposed drugs could enhance the effect of chemotherapy, we first performed a cell apoptosis assay in attached cell lines (MDA-MB-231 and SUM159) at 24 h of treatment. As depicted in Fig. S8C, these combinations (D+E, D+O, D+V) enhanced the apoptotic population compared to D (5 nM) and their respective monotherapies with each repurposed drug. Accordingly, we next analyzed the behavior of these combinations on the cell viability in the UGR01 PDxOs model. At the density of PDxOs analyzed, contrary to D, E, and O, it was found that monotherapy with V reduced cell viability (**** P < 0.0001) compared to vehicle control (Fig. 5G). Nonetheless, we found that the viability of organoids was compromised when the repurposed drugs were combined with docetaxel: D+E (** P < 0.01), D+O, and D+V (**** P < 0.0001) (Fig. 5G).

### Repurposed drugs are effective in docetaxel-resistant cell lines

**Figure 6:**
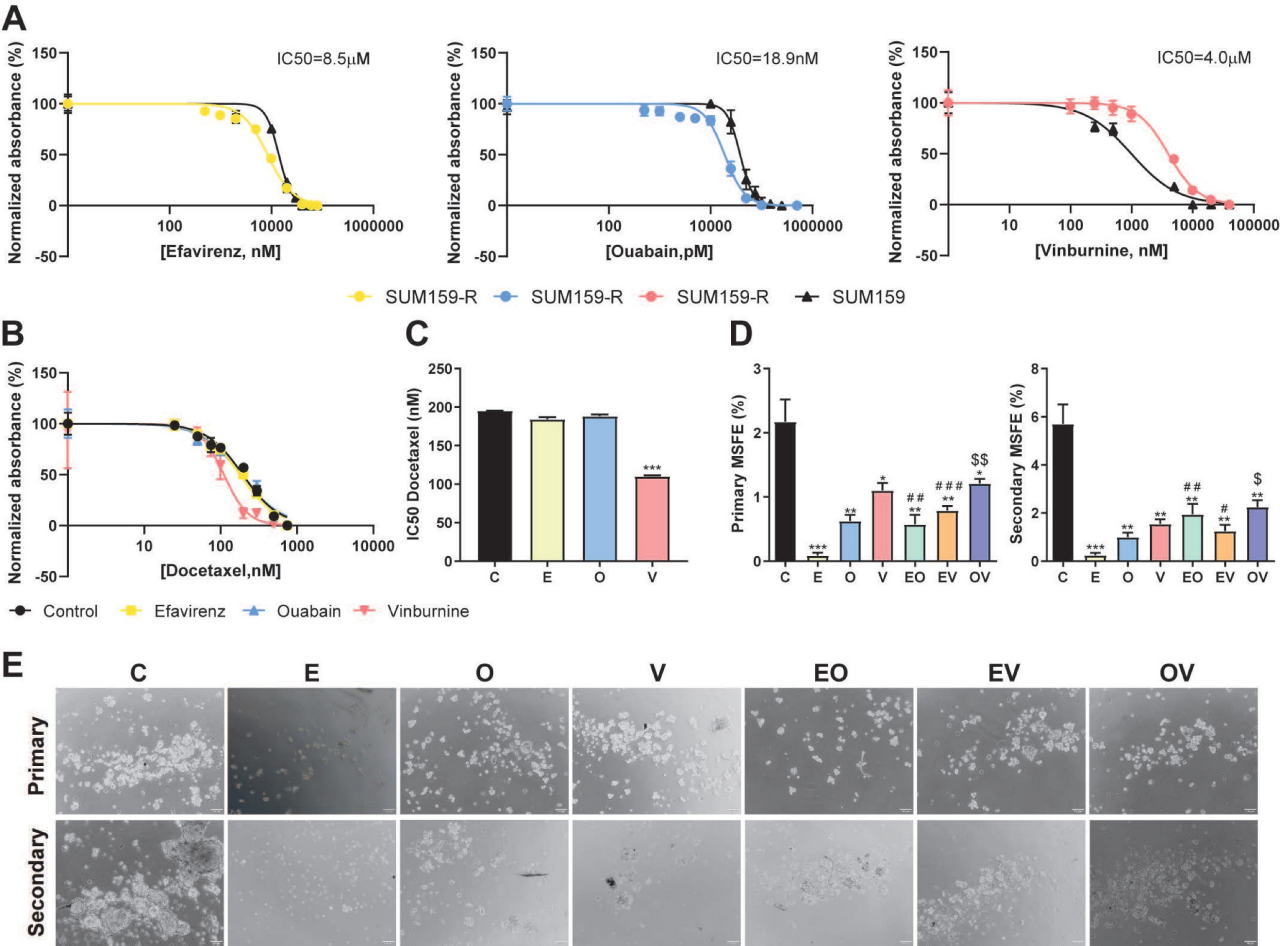
** Repurposed drugs are effective in docetaxel-resistant TNBC cell line.** (A) IC50 of Efavirenz (E), Ouabain (O), and Vinburnine (V) in SUM159-R and SUM159 cell lines after 72-h treatments. The black line represents SUM159, and the colored lines represent the SUM159-R cell line. Results were normalized to the vehicle control. Data are presented as mean ± SD (n=6). (B) IC50 sigmoid curve of Docetaxel after 72-h treatments in combination with vehicle control or repurposed drugs. (C) Changes in the IC50 of Docetaxel when combined with the repurposed drugs or vehicle as control. Data are presented as mean ± SD (n=2). (D) Mammosphere-forming efficiency (MSFE) in primary and secondary mammospheres after 72-h treatments in the SUM159-R cell line. Data are presented as mean ± SD (n=4) (E) Representative images of primary and secondary generations of mammospheres of SUM159-R cell line. Statistically significant differences with the vehicle control (C): * P ≤ 0.05, ** P < 0.01, *** P <0.001. Statistically significant differences with E: # P ≤ 0.05, ## P < 0.01, ### P < 0.001. Statistically significant differences with O: $ P ≤ 0.05, $$ P < 0.01.

To enhance the value of our repurposed drugs and their combination with the taxane docetaxel, we study their effectiveness in our previously generated docetaxel-resistant cell line SUM159-R^20^. First, Fig. 6A illustrates that SUM159-R exhibited distinct sensitivity profiles compared to the parental SUM159 cells. Notably, SUM159-R displayed enhanced sensitivity to E and O, with IC50 values of 8.5 µM and 18.9 nM, respectively. Conversely, these cells demonstrated increased resistance to V, with an IC50 of 4.0 µM. Further, we assess if our repurposed drugs could modulate D sensitivity and potentially reverse chemoresistance (Fig. 6B, 6C). Interestingly, E and O did not significantly alter docetaxel sensitivity as we calculated IC50 values of 182 nM and 196 nM, respectively, while the vehicle control presented an IC50 of 196 nM. However, co-treatment with V resulted in a marked reduction in the IC50 of D (112 nM) compared to vehicle (*** P < 0.001, Fig. 6C). Lastly, motivated by our previous findings against CSCs, we assessed the impact of our repurposed treatments on the self-renewal capacity of SUM159-R cells. As depicted in Fig. 6D and 6E, all treatments effectively reduced both primary and secondary MSFE. Notably, E monotherapy emerged as the most efficacious in suppressing self-renewal, followed by O. This consistent suppression of self-renewal capacity across all treatments underscores the promise of these repurposed drugs in targeting cancer stem cell-like properties in chemoresistant TNBC.

## Discussion

In patients with TNBC, conventional chemotherapy stands as the primary therapeutic approach. However, drug resistance, metastasis, and recurrence predominantly arise from the subpopulation of CSCs^4^. Despite recent approvals of novel therapies for metastatic TNBC^2^, the need for further targeted interventions persists due to its significant heterogeneity^4^. While developing new drugs is a lengthy endeavor, repurposing FDA-approved medications offers a more efficient alternative, requiring less time and fewer resources. This approach has been exemplified by successful cases in TNBC, such as anthracyclines (doxorubicin, epirubicin) and gemcitabine^8^. Our previous work elucidated that the TGFβ pathway modulates the aggressiveness of NDRG1 in TNBC cell lines and demonstrated that its inhibition after TGFβ stimulation leads to diminished expression of EMT markers, CSCs, and migration^5^. Consequently, we currently propose a drug repurposing study based on the gene expression signature of these conditions, thus circumventing potential complications linked to the direct inhibition of pleiotropic NDRG1 protein^5^,^32^.

While most researchers followed the "gene reversion" technique, namely the study of drug candidates capable of reversing the gene expression patterns of a given disease^33^,^34^, we aimed to replicate the antitumor transcriptional characteristics observed upon *NDRG1* inhibition following TGFβ stimulation using CMap^10^. In the results of PCL analysis, the presence of MAPKs, WNT family, and histone-modifying EMSY complex loss of function, and the expression of TGFβ receptor inhibitors support the hypothesis that drugs based on their similarity to *NDRG1* knockdown profile may exert an anti-tumorigenic effect^35^-^38^. Moreover, ATPase inhibitors (also known as cardiac glycosides) have garnered significant attention for their potential in various types of cancer, showcasing promising outcomes^39^, including breast cancer^40^, among others. Interestingly, these inhibitors are implicated in orchestrating diverse forms of cell death, such as apoptosis, autophagy, and immunogenic cell death^39^. Consequently, their classification as the best disruptive agents in the *General*, *Summary,* and MCF-7 categories underscored the considerable potential of these compounds (Fig. 1B). Nonetheless, a limitation of the CMap platform is that MCF7 is the only breast cancer cell line included, being an estrogen receptor-positive (ER+) subtype, thus not representative of the TNBC subtype. This may raise concerns regarding the specificity of the identified compounds for TNBC. However, previous studies have shown that MCF7 cells can partially mimic TNBC behavior in certain contexts, including metabolic profiles and signaling activity^41^,^42^. This partial overlap may help explain the predictive value of the CMap results in our TNBC models and support the translational potential of the identified compounds.

Given the complexity of this tool, several categories of results were considered for drug selection, and we performed two queries to compare the similarities among the results. As described previously, several drugs with high connectivity scores in the ±1.5 query did not appear in the ±1.6 query. Hence, this absence suggests that the connectivity of those drugs was primarily based on genes with a ±1.5 log_2_ fold change, which likely had a lesser contribution to the desired outcome. Consequently, we chose three candidates from the ±1.6 results for our study: efavirenz, a non-nucleoside reverse transcriptase inhibitor used for the treatment of HIV infection that has previously been analyzed in TNBC^43^,^44^; ouabain, an ATPase inhibitor that has been reported as a potential treatment for various types of cancer such as lung^45^ and breast cancer^45^,^46^; and vinburnine (also known as eburnamonine), a vasodilating alkaloid and allosteric modulator of muscarinic acetylcholine receptors^47^ with antiproliferative effects in leukemia^48^. To support drug repurposing^8^,^9^, all the compounds selected herein were FDA-approved and presented as novel options for the clinical treatment of TNBC.

To validate their effectiveness, we studied cell viability and successfully obtained IC50 values within the comparable range documented by previous researchers^44^,^46^,^49^. Furthermore, other researchers demonstrated the lower toxicity of E and O in an immortalized normal breast tissue cell line (MFC-10A)^43^,^46^, supporting the potential of these treatments. Given the significant deficiency in studies that combine repurposed drugs in cancer research, and to untangle the possibility of synergistic effects, we analyzed combinations of E, O, and V to ascertain whether they could exhibit enhanced anticancer efficacy compared to monotherapies. We also observed increased cell apoptosis in the three cell lines tested. It is known that the higher abundance of CSCs in TNBC, compared with other breast cancer subtypes, is responsible for its aggressiveness^24^. Regarding the SUM159 cell line, better results were obtained in the ALDH1+ CSC subpopulation, where all treatments strongly reduced it, except for E monotherapy. Whereas in BT549 cells, E, O, EO, and EV were the therapies that decreased ALDH1+ and CD44^high^/CD24^-^ populations. While in SUM159 and BT549 cell lines, there are no previous reports of the impact of our repurposed drugs in CSC subpopulations, E therapy was tested in MDA-MB-231 cells^44^. Our data revealed that all treatments led to a marked reduction in the metastatic, chemoresistant, and proliferative ALDH1+ population^24^ in MDA-MB-231 cells, where, specifically, the decrease obtained after E treatment concorded with the findings of Chiou *et al.*^44^. Regarding the invasive CD44^high^/CD24^-^ population^24^, while treatment responses varied between cell lines, the best results were again in MDA-MB-231, with significant decreases in the CD44^high^/CD24^-^ population across all treatment groups. Chiou *et al.* did not observe differences with E in the CD44^+^/CD24^-^ subpopulation, while we noted a reduction in the CD44^high^/CD24^-^ subpopulation in this cell line^44^. Preliminary results of the effects of treatments in the side population, a residual subpopulation characterized by drug resistance^50^, showed a decrease with all treatments except in MDA-MB-231 with EV and OV.

Collectively, our data indicate that the repurposed drugs and their combinations demonstrate significant efficacy in reducing diverse CSC populations. However, we found a better response in MDA-MB-231 cells, which could be explained by the fact that it is the cell line used for the RNA-seq study, and the intrinsic molecular heterogeneity among the TNBC cell lines tested in our study, which could contribute to the differential drug responses observed across the models^51^. The reduction in CSC subpopulations, coupled with the overall decrease in MSFE, colony formation, and cellular migration, provides robust evidence for the anticancer activity of our repurposed drug candidates. Interestingly, EO did emerge as the most potent therapy, significantly reducing nearly all CSC phenotypic properties.

Regarding the mechanism of action of these repurposed candidates, we did not observe the compounds inhibiting NDRG1, similar to other researchers who used CMap and found the same outcome^52^,^53^. Additionally, our previous findings suggest that not all TNBC patients may have active TGFβ-driven tumorigenic NDRG1, where NDRG1 could act as a tumor suppressor, making its inhibition undesirable in those patients^5^. Therefore, these drug candidates cannot be considered NDRG1 inhibitors, thus avoiding any negative effect derived from the direct inhibition of the antitumor version of NDRG1. Instead, we assessed other targets such as CXCL10 and CXCL11, which activate the AKT pathway^27^ and observed reduced phosphorylation of this protein upon treatment. For instance, previous investigations with E treatment in pancreatic cancer^54^ and with O therapy in lung^55^ cancer did demonstrate the same alteration of the pathway. Furthermore, PODXL is involved in EMT through the PI3K/AKT pathway^26^, and OAS1 is an EMT-related protein modulated by AKT^29^, consequently, the reduction in EMT markers observed by western blot can be expected after repurposed treatments. These results align with observations of altered pathways potentially responsible for anticancer properties. Particularly, E reduces the expression of LINE-1 (long interspersed nuclear element 1) retrotransposon, which is overexpressed in TNBC cell lines^43^, O reduces STAT3 signaling in lung and cervical cancer^56^, and V seems to exert its cytotoxic effects via oxidative stress pathways^48^. In summary, these findings support the anticancer activity exerted by our repurposed drugs, with a significant reduction of CSC phenotypic properties and migration (Fig. 3). Taking knowledge of the relationship between the AKT pathway and CSCs^57^, the fact that EO was the most potent combination of reducing stemness markers corroborates with the decrease in AKT and EMT proteins (Fig. 4B and 4C).

To increase the robustness of the validation of our candidates, we used a well-established model of PDxO derived from a TNBC patient that mirrors the intrinsic characteristics of the original breast tumor, as expected^15^, with minimal murine presence, consistent with other studies^22^. Besides, our patient presented high basal expression of NDRG1 in both the PDX and PDxO model, which correlates with poorer survival of TNBC patients^5^. Our three candidates reduced the expression of the proliferation marker Ki67^30^ and p-AKT, corroborating the mechanism pathway of the drugs that we observed in the attached cell lines. Furthermore, combinations with the taxane D demonstrated lower cell viability than the monotherapies, suggesting the potential for a synergistic effect, which supports their use as co-adjuvants in TNBC treatment. However, a key limitation of PDxOs is the absence of immune and stromal components, which restricts their ability to fully recapitulate tumor-immune interactions. Although efforts like immune cell co-cultures or preservation of tumor-infiltrating lymphocytes are being explored, these remain technically challenging and not yet standardized^58^,^59^.

Because chemoresistance remains a critical obstacle in TNBC therapy^60^, we assessed the repurposed drugs in docetaxel-resistant SUM159-R cells^20^. Our analysis revealed increased sensitivity to E and O, supporting the concept of collateral sensitivity^60^. Whereas V treatment demonstrated the ability to restore D efficacy in resistant cells, likely by targeting drug efflux pumps and CSC-related populations such as ALDH1+ and CD44^high^/CD24^24^. Additionally, all three repurposed drugs significantly reduced MSFE in the chemoresistant line, suggesting their potential to target CSCs linked to treatment resistance^60^. These results propose two promising strategies for second-line TNBC treatment: monotherapy with E or O, or combination therapy with D+V. Further studies are needed to validate these promising approaches.

## Conclusion

In this study, we present a comprehensive approach, starting with computational research, computational research, progressing to the research in two-dimensional cell models of TNBC, and culminating in validation in a TNBC patient-derived 3D organoid cultures that mimic the patient´s tumor characteristics, which strengthen our results and allow the translation from bench to clinic. In conclusion, our study reveals that repurposed drugs, based on the transcriptomic signature of inhibition of *NDRG1* under TGFβ1 stimulation, present potential effectiveness for TNBC treatments. These findings offer viable effective therapeutic strategies and pave the way for developing innovative therapies for TNBC.

## Supplementary Material

Supplementary figures and tables.

## Figures and Tables

**Table 1:** Selection of repurposed drugs.

Drug	Description	Relate disease	Connectivity score
Efavirenz	HIV reverse transcriptase inhibitor	HIV (infectious disease)	Summary / General 98.98 / 93.63
Ouabain	ATPase inhibitor	Hypertension, arrhythmia	MCF-7 99.17
Vinburnine	Allosteric modulator of muscarinic acetylcholine receptors	Cerebral stroke	Summary / General 97.57 / 93.53

HIV: human immunodeficiency virus.

**Table 2:** IC50 values and selected treatment doses for drug candidates.

Drug	IC50 value			Treatment dose		
	MDA-MB-231	SUM159	BT549	MDA-MB-231	SUM159	BT549
Efavirenz (µM)	15.4	13.9	1.6	15	15	2
Ouabain (nM)	54.0	38.0	22.7	54	38	23
Vinburnine (µM)	1.0	1.0	5.6	1	1	6

## Abbreviations

ALDH1: aldehyde dehydrogenase 1
CMap: Connectivity Map
CSCs: cancer stem cells
D: docetaxel
DEAB: diethylaminobenzaldehyde
DMEM: dulbecco's modified eagle medium
E: efavirenz
EGF: epidermal growth factor
EMT: epithelial-mesenchymal transition
EO: efavirenz + ouabain
ER: estrogen
EV: efavirenz + vinburnine
FBS: fetal bovine serum
FISH: fluorescence in situ hybridization
IC50: half maximal inhibitory concentration
MAPKs: mitogen-activated protein kinases
MHC: Major Histocompatibility Complex
MGMT: O6-methylguanine-DNA methyltransferase
MSFE: mammosphere-forming efficiency
NDRG1: N-myc downstream gene 1
O: ouabain
OV: ouabain + vinburnine
PCL: perturbagen classes
PDxOs: patient-derived xenograft organoids
PDX: patient-derived xenograft
PFA: paraformaldehyde
PI: propidium iodide
PR: progesterone
qPCR: quantitative PCR
RNA-seq: RNA sequencing
RSEM: RNA-seq by expectation maximization
RT: room temperature
SD: standard deviation
TNBC: triple-negative breast cancer
V: Vinburnine
