# Supplementary material for: Signature-based repurposed drugs resemble the inhibition of TGFβ-induced NDRG1 as potential therapeutics for triple-negative breast cancer
Source: Int J Biol Sci. 2025 Jun 9;21(9):3949–67. doi: 10.7150/ijbs.112645 (PMC12223764; doi:10.7150/ijbs.112645)
Supplement: Supplementary file 1 — Supplementary figures and tables. [file ijbsv21p3949s1.pdf]

Supplementary material

Signature-based repurposed drugs resemble the inhibition of TGFβ-induced NDRG1 as potential therapeutics for triple-negative breast cancer.

Araceli López-Tejada[1,2,3,#], Jose L. Blaya-Cánovas[2,3,4,#], Francisca E. Cara[3], Jesús Calahorra[2,3,4], César Ramírez-Tortosa[3,5], Isabel Blancas[3,6,7], Violeta Delgado-Almenta[2], Fabiola Muñoz-Parra[8], Marta Ávalos-Moreno[2], Ana Sánchez[2], Adrián González-González[2], Juan A. Marchal[3,9,10,11], Carmen Griñán-Lisón[1,2,3,11,*], Sergio Granados-Principal[1,2,3,*].

[1]Department of Biochemistry and Molecular Biology II, Faculty of Pharmacy, University of Granada, Campus de Cartuja s/n, Granada, Spain

[2]GENYO, Centre for Genomics and Oncological Research, Pfizer/University of Granada/Andalusian Regional Government, Granada, Spain

[3]Instituto de Investigación Biosanitaria ibs.GRANADA, Granada, Spain

[4]UGC de Oncología Médica, Hospital Universitario de Jaén, Jaén, Spain

[5]UGC de Anatomía Patológica, Hospital Universitario "San Cecilio", Granada, Spain

[6]UGC de Oncología, Hospital Universitario "San Cecilio", Granada, Spain

[7]Department of Medicine, University of Granada, Granada, Spain

[8]UGC de Radiodiagnóstico, Hospital Universitario "San Cecilio", Granada, Spain

[9]Biopathology and Regenerative Medicine Institute (IBIMER), Centre for Biomedical Research (CIBM), University of Granada, Granada, Spain

[10]Department of Human Anatomy and Embryology, Faculty of Medicine, University of Granada, Granada, Spain

[11]Excellence Research Unit "Modeling Nature" (MNat), Centre for Biomedical Research (CIBM), University of Granada, Granada, Spain

[#]These authors contributed equally.

[*]Corresponding authors: carmengl@go.ugr.es (Carmen Griñán-Lisón), sergiogp@ugr.es (Sergio Granados-Principal). Tel.: +34 651557921

Supplementary Figures

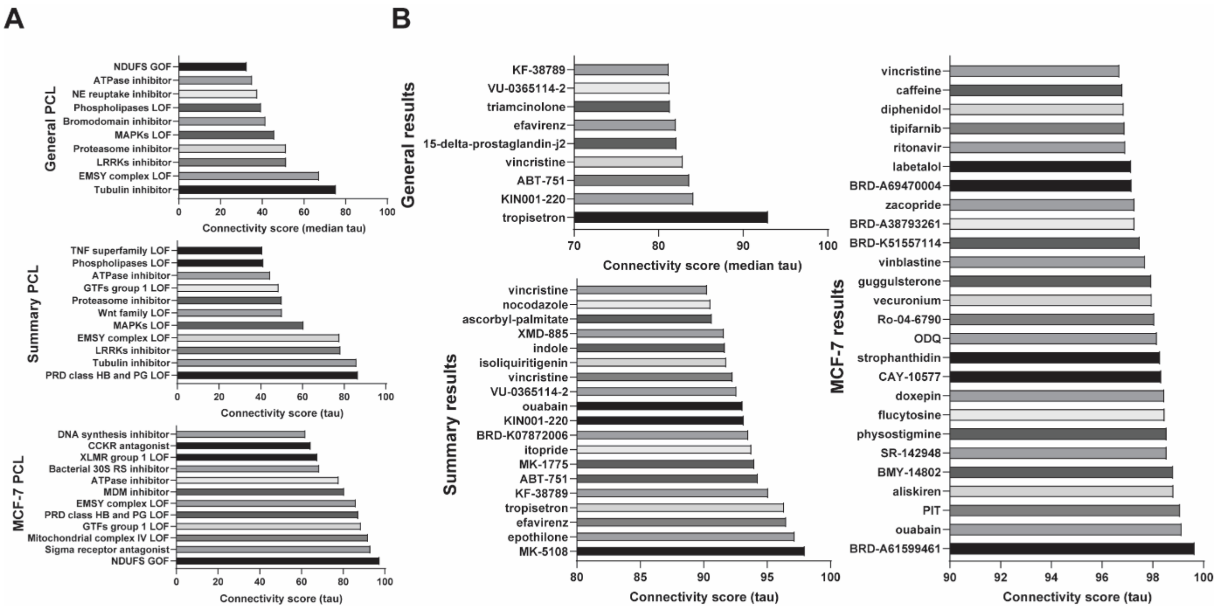

Fig. S1. CMap results of ±1.5 $\log_2$ fold-change query. (A) Perturbagen class (PCL) topmost connectivity scores; (B) Compounds with connectivity scores higher than 80, 90, and 96 for General, Summary, and MCF-7 cell line analyses, respectively. CCKR, Cholecystokinin Receptor; GOF, Gain Of Function; GTFs, General Transcription Factors; HB, Homeoboxes; LOF, Loss Of Function; LRRKs, Leucine Rich Repeat Kinases; MAPKs, Mitogen Activated Protein Kinases; MDM, Murine Double Minute; NDUFS, NADH ubiquinone oxidoreductase core subunits; NE, Norepinephrine; PG, Pseudogenes; RS, ribosomal subunit; TNF, Tumor Necrosis Factor; XLMR, X-Linked Mental Retardation.

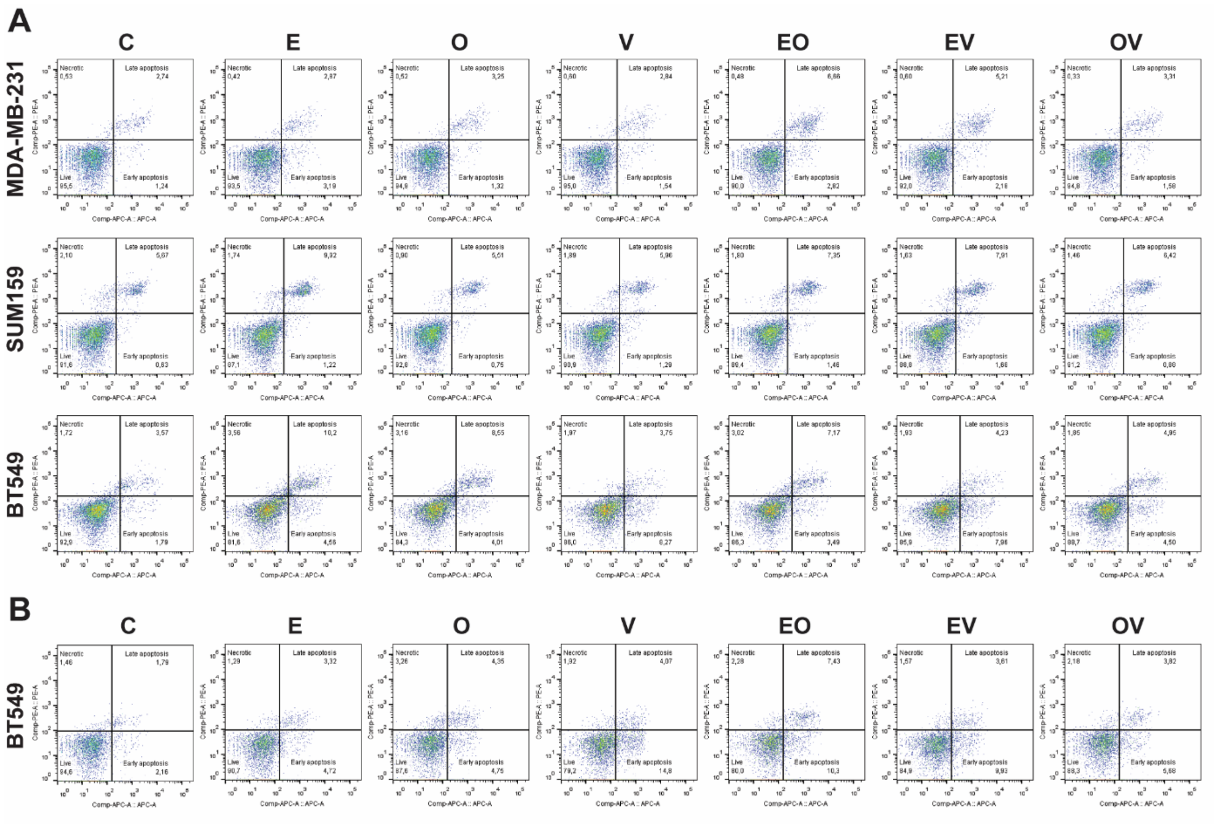

Fig. S2. (A) Representative dot plots of Annexin V/PI FACS analysis: viable cells (Annexin V and PI negative cells); early apoptotic cells (Annexin V positive and PI negative cells); late apoptotic cells (Annexin V and PI positive); and necrotic cells (Annexin V negative and PI positive), in MDA-MB-231, SUM159 and BT549 cell lines after 24-hour treatments; (B) Representative dot plots of Annexin V/PI FACS analysis in BT549 cell line after 48-hour treatments.

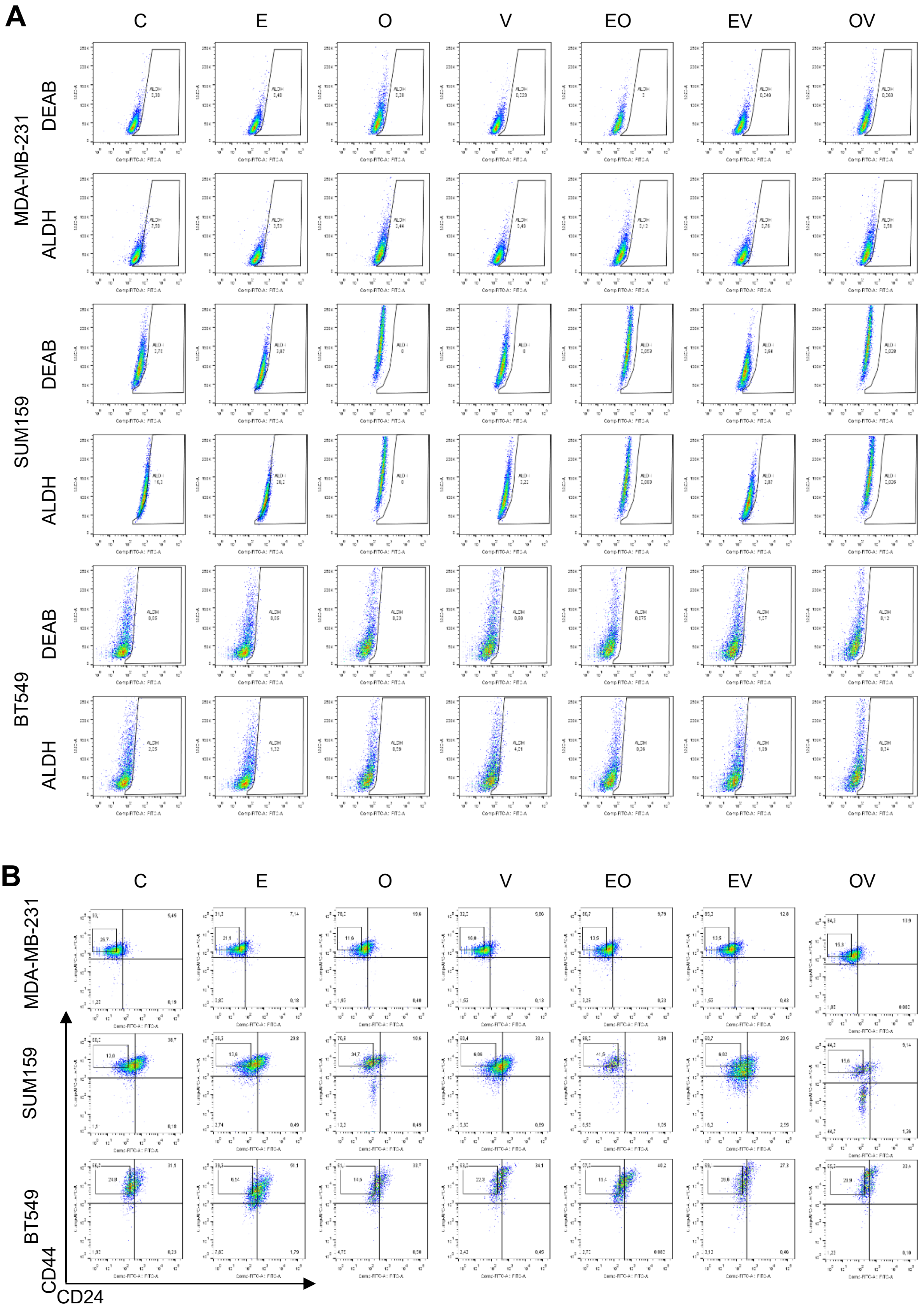

Fig. S3. (A) Representative dot plots of flow cytometric analysis of aldefluor-positive (ALDH) population and DEAB control after 72-hour treatments in MDA-MB-231, SUM159, and BT549

cell lines. (B) Representative dot plots of flow cytometric analysis of CD44^{high}/CD24^{-} population in MDA-MB-231, SUM159, and BT549 cell lines after 72-hour treatments.

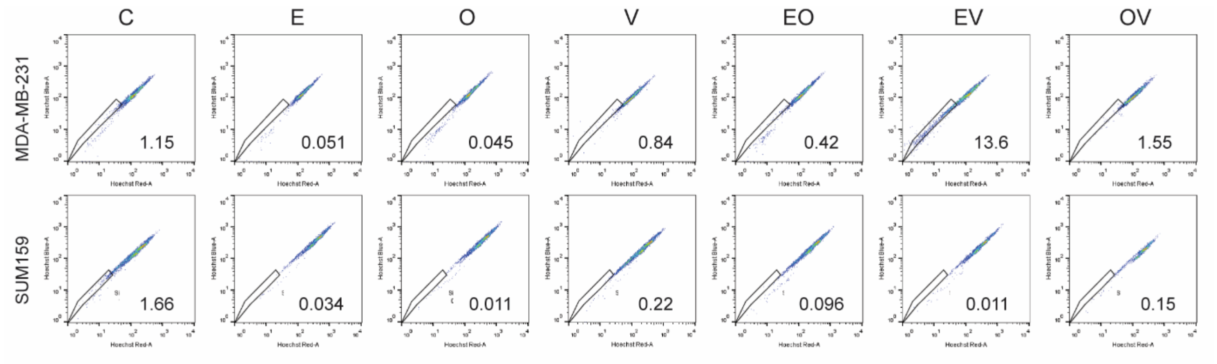

Fig. S4. Dot plots of side population flow cytometry after 72-hour treatments in MDA-MB-231 and SUM159.

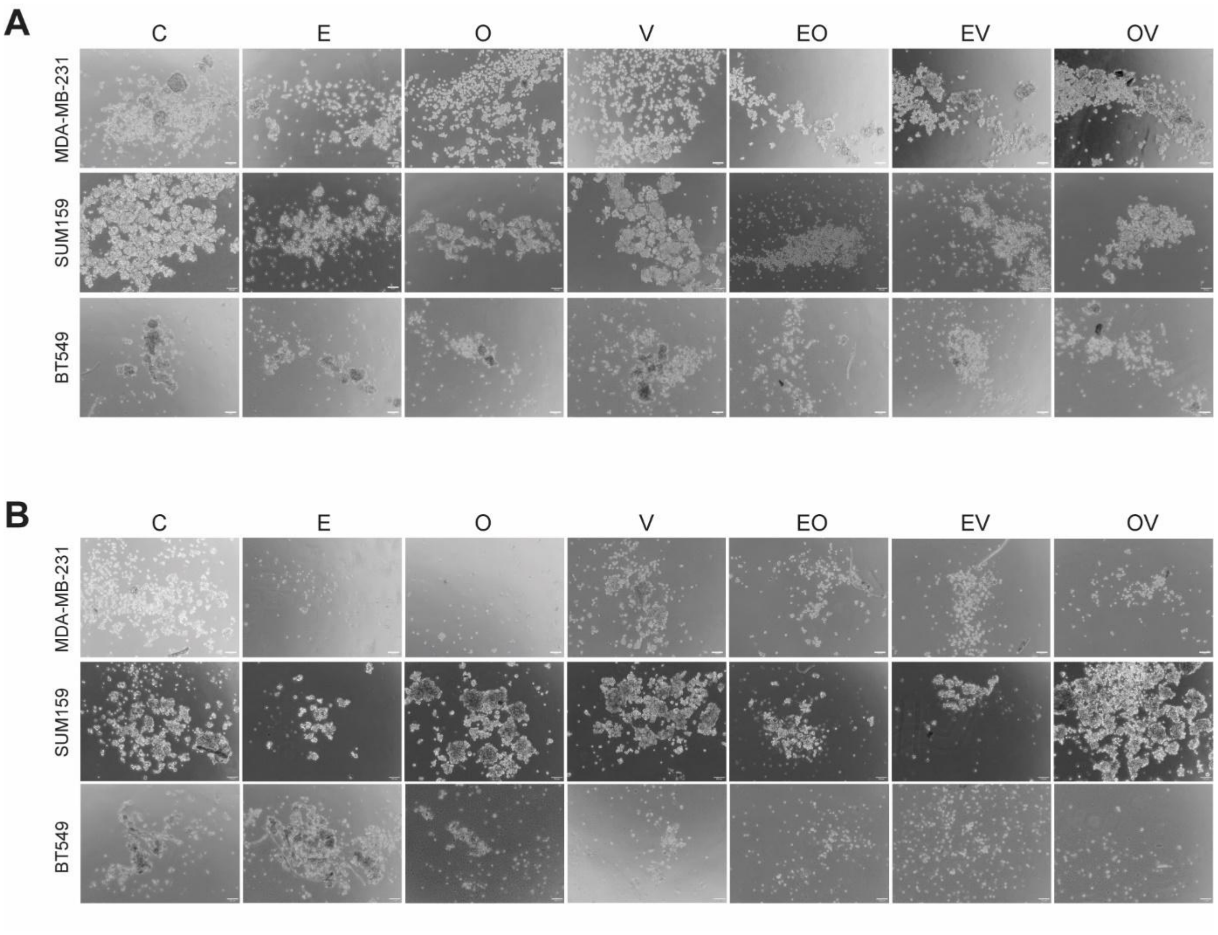

Fig. S5. (A) Representative images of primary and (B) secondary generations of mammospheres of MDA-MB-231, SUM159, and BT549 cell lines.

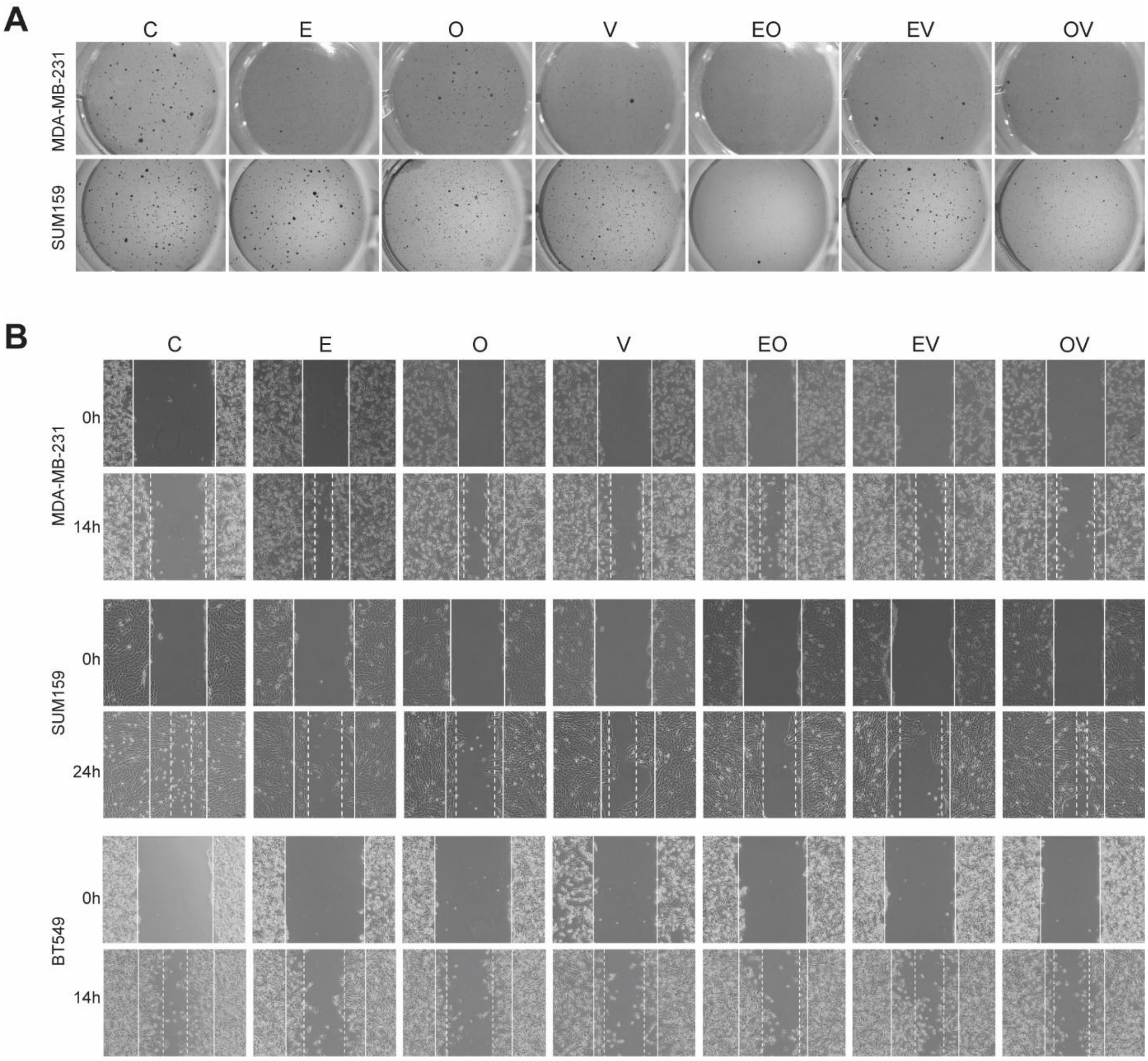

Fig. S6. (A) Representative images of soft agar colony formation of MDA-MB-231 and SUM159 cell lines. (B) Representative images of tumor cell migration after 24-hour treatments in MDA-MB-231, SUM159, and BT549 cell lines.

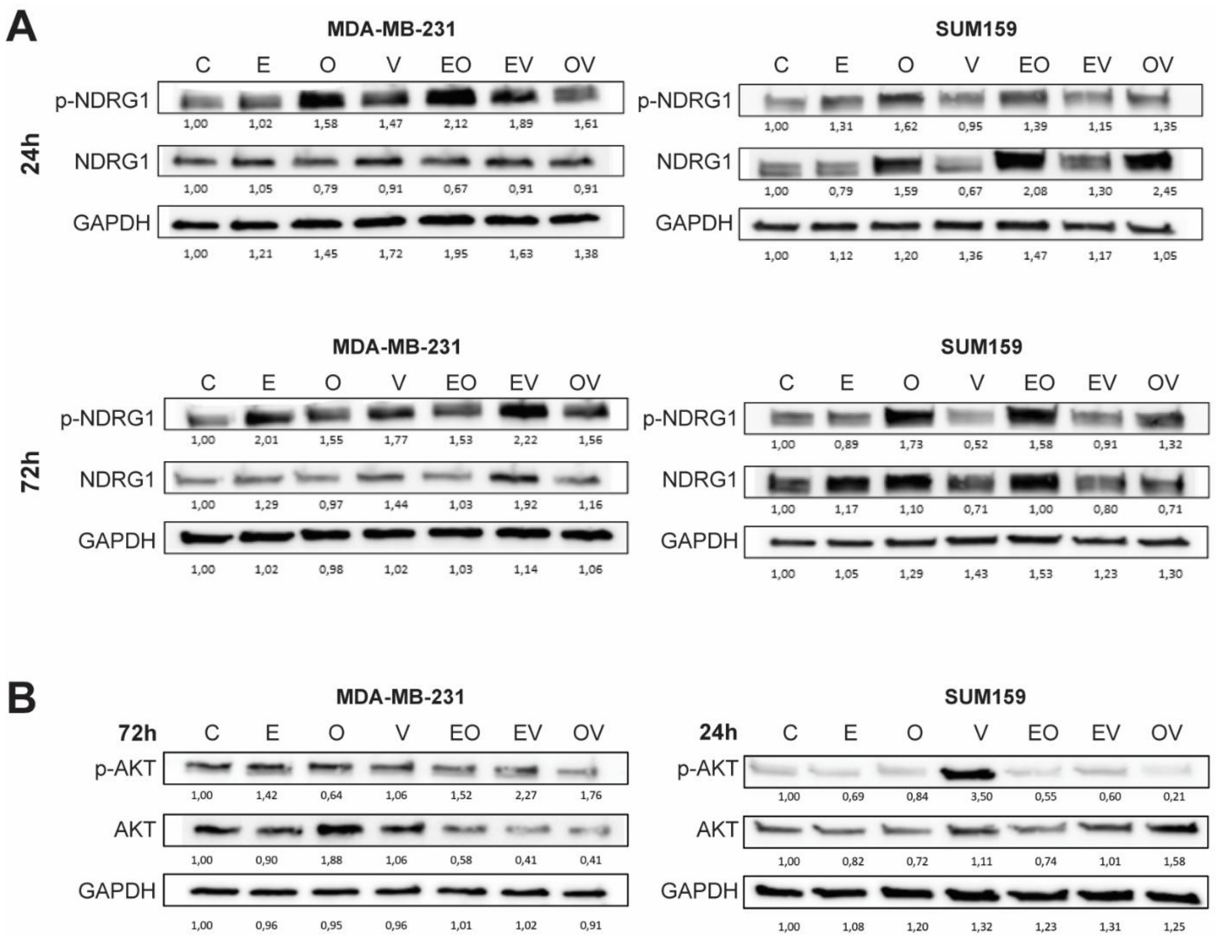

Fig. S7. (A) Western blot and densitometric analysis of p-NDRG1 (Thr346) and total NDRG1 after 24 and 72-hour treatments in MDA-MB-231 and SUM159 cell lines. (B) Western blot and densitometric analysis of p-AKT (Ser473) and total AKT after 72-h treatments in MDA-MB-231 and after 24-h SUM159 cell lines.

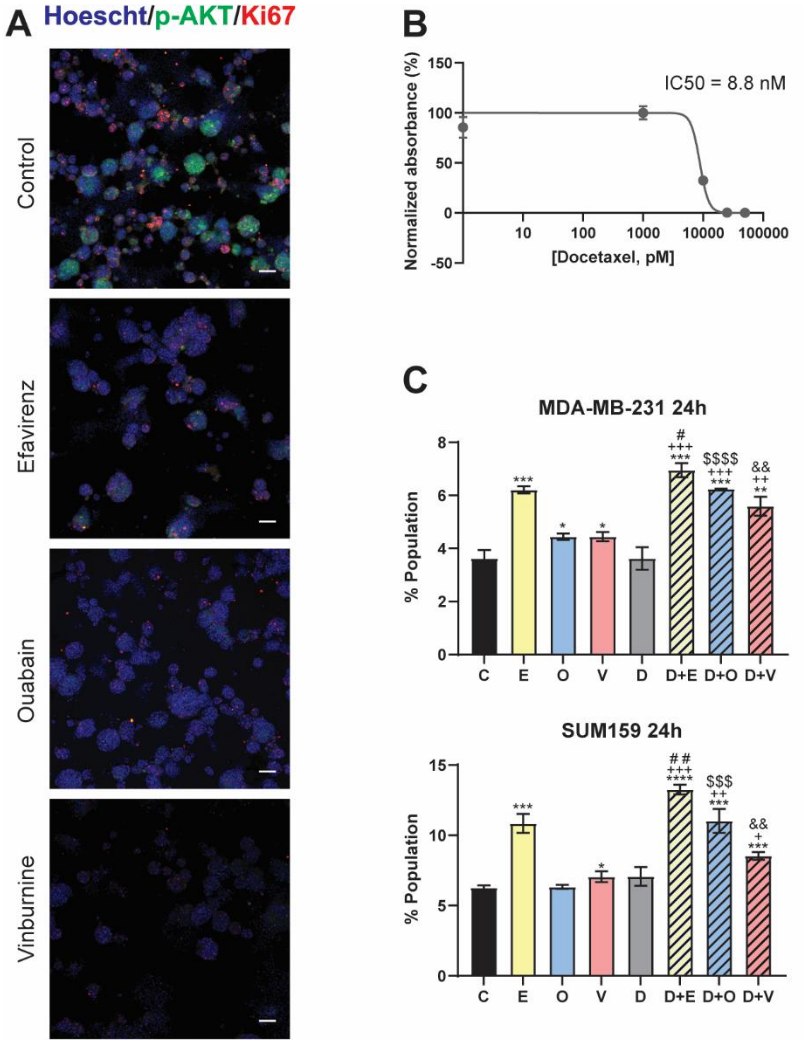

Fig. S8. (A) Representative confocal images (original optical objective: 10x) of p-AKT (green) and Ki67 (red) in UGR01 PDxOs after 72-hour treatments. Scale bar 100 µm. (B) IC50 of Docetaxel (D) in the UGR01 PDxOs model after 72-hour treatments. Results were normalized to the vehicle control (n=5). (C) Flow cytometric analysis of total apoptotic population after 24-hour treatments in MDA-MB-231 and SUM159 cell lines (n=3). Data are presented as mean ± SD. Statistically significant differences with the vehicle: * P < 0.05, ** P < 0.01, *** P < 0.001, **** P < 0.0001. Statistically significant differences with docetaxel: + P < 0.05, ++ P < 0.01, +++ P < 0.001. Statistically significant differences with E: # P < 0.05, ## P < 0.01. Statistically significant differences with O: $$$ P < 0.001, $$$$ P < 0.0001. Statistically significant differences with V: && P < 0.01.

Supplementary Tables

Table S1. Transcriptomic profile from NDRG1 knockdown in TGFβ1-treated MDA-MB-231 cells compared to stimulation with TGFβ1 used for the clue.io query.

Gene Stable ID	Gene name	$\log_2$ FoldChange	p-value	p-adjusted	Included
ENSG00000077984	*CST7*	2,891200479	4,0778E-08	9,8694E-07	Valid
ENSG00000127884	*ECHS1*	2,452566431	3,477E-66	2,9033E-63	Valid
ENSG00000172667	*ZMAT3*	2,298752408	7,1603E-41	2,6157E-38	Valid
ENSG00000134602	*STK26*	2,233857126	7,9786E-09	2,1998E-07	Valid
ENSG00000187068	*C3orf70*	2,206731586	3,1769E-05	0,00040063	Invalid
ENSG00000272916	*AC022400.6*	2,171647427	5,9098E-06	9,0072E-05	Invalid
ENSG00000269028	*MTRNR2L12*	2,160385347	0,0042228	0,02434151	Invalid
ENSG00000171241	*SHCBP1*	2,039301485	4,0875E-54	2,2754E-51	Valid
ENSG00000167081	*PBX3*	2,029327334	8,833E-31	1,8774E-28	Valid
ENSG00000006576	*PHTF2*	1,949970154	8,5491E-38	2,701E-35	Valid
ENSG00000114125	*RNF7*	1,939075104	3,4133E-34	9,0685E-32	Valid
ENSG00000177432	*NAP1L5*	1,913948912	0,00011559	0,00121963	Valid but not used
ENSG00000184992	*BRI3BP*	1,861301075	1,1925E-23	1,7872E-21	Valid but not used
ENSG00000163734	*CXCL3*	1,804699297	1,5057E-05	0,0002083	Valid
ENSG00000186517	*ARHGAP30*	1,791681042	0,00036863	0,00327951	Valid but not used
ENSG00000197296	*FITM2*	1,79166835	2,1074E-13	1,1959E-11	Valid but not used
ENSG00000180758	*GPR157*	1,727575436	2,2153E-13	1,2421E-11	Valid
ENSG00000064666	*CNN2*	1,718786337	1,3853E-43	5,7836E-41	Valid
ENSG00000163378	*EOGT*	1,684725681	4,2987E-26	7,1788E-24	Valid
ENSG00000108468	*CBX1*	1,670397289	1,8655E-44	9,0863E-42	Valid
ENSG00000095752	*IL11*	1,668333067	2,5895E-85	4,3245E-82	Valid
ENSG00000197111	*PCBP2*	1,662640882	1,141E-43	4,94E-41	Valid
ENSG00000114450	*GNB4*	1,654412255	1,3032E-31	2,9872E-29	Valid but not used
ENSG00000185787	*MORF4L1*	1,634487115	2,8458E-70	2,7723E-67	Valid
ENSG00000188167	*TMPPE*	1,633461654	2,1789E-10	7,8372E-09	Valid but not used

ENSG00000274290	*HIST1H2BE*	1,625233997	5,3831E-08	1,2769E-06	Valid but not used
ENSG00000092820	*EZR*	1,613883538	3,4291E-63	2,6724E-60	Valid
ENSG00000179598	*PLD6*	1,611512464	0,00024874	0,00234874	Valid but not used
ENSG00000185129	*PURA*	1,567471598	3,9245E-32	9,3627E-30	Valid
ENSG00000123685	*BATF3*	1,560864209	0,00307458	0,01900679	Valid
ENSG00000188706	*ZDHHC9*	1,558074131	1,0907E-10	4,0999E-09	Valid but not used
ENSG00000184743	*ATL3*	1,553878289	1,1899E-70	1,2645E-67	Valid but not used
ENSG00000170540	*ARL6IP1*	1,54432197	1,4697E-92	4,2952E-89	Valid
ENSG00000152749	*GPR180*	1,533783667	8,9653E-16	6,85E-14	Valid but not used
ENSG00000114999	*TTL*	1,528151806	8,4473E-39	2,9924E-36	Valid but not used
ENSG00000166471	*TMEM41B*	1,520338217	2,2394E-43	9,0272E-41	Valid
ENSG00000009335	*UBE3C*	1,517270352	1,0684E-87	2,0815E-84	Valid
ENSG00000147676	*MAL2*	-1,519193404	1,6147E-11	6,8144E-10	Valid but not used
ENSG00000283378	*BX088645.1*	-1,530831953	0,00332468	0,02025048	Invalid
ENSG00000109113	*RAB34*	-1,532075162	3,6608E-56	2,3775E-53	Valid but not used
ENSG00000164100	*NDST3*	-1,537049346	0,00205093	0,01377895	Valid
ENSG00000131781	*FMO5*	-1,571411849	0,00410901	0,02379117	Valid
ENSG00000112697	*TMEM30A*	-1,579677089	1,9091E-71	2,2318E-68	Valid
ENSG00000179546	*HTR1D*	-1,584293556	7,7899E-05	0,00087057	Valid
ENSG00000167578	*RAB4B*	-1,63264198	3,8364E-05	0,00047208	Valid
ENSG00000111731	*C2CD5*	-1,646571795	2,743E-22	3,6029E-20	Valid
ENSG00000168702	*LRP1B*	-1,647442934	0,00035193	0,0031477	Valid
ENSG00000089127	*OAS1*	-1,659312066	0,00128731	0,00939957	Valid
ENSG00000133135	*RNF128*	-1,688883665	3,9904E-08	9,698E-07	Valid
ENSG00000142634	*EFHD2*	-1,716628773	1,1323E-23	1,719E-21	Valid
ENSG00000165806	*CASP7*	-1,781981672	1,2152E-16	1,0445E-14	Valid
ENSG00000170004	*CHD3*	-1,889719302	1,1782E-50	6,2606E-48	Valid
ENSG00000132842	*AP3B1*	-1,950059614	1,18E-89	2,7587E-86	Valid
ENSG00000128567	*PODXL*	-2,040318675	2,1247E-38	7,0966E-36	Valid

ENSG00000164023	*SGMS2*	-2,07587609	2,5007E-44	1,1693E-41	Valid but not used
ENSG00000170801	*HTRA3*	-2,228843042	0,00011798	0,00124358	Valid but not used
ENSG00000169248	*CXCL11*	-2,366645215	3,1104E-09	9,2051E-08	Valid
ENSG00000104419	*NDRG1*	-2,79571715	1,472E-143	1,721E-139	Valid
ENSG00000138135	*CH25H*	-2,925202949	2,0057E-06	3,4129E-05	Valid
ENSG00000169245	*CXCL10*	-3,09717104	6,756E-18	6,7502E-16	Valid
ENSG00000257411	*AC034102.2*	-6,753130759	0,00751658	0,03882848	Invalid
ENSG00000256514	*AP003419.1*	-6,985214008	4,8665E-05	0,00057752	Invalid

Invalid: Not a valid HUGO symbol or Entrez ID, not used in the query.

Valid: Valid HUGO symbol or Entrez ID and part of the BING space, used in the query.

Valid but not used: Valid HUGO symbol or Entrez ID not part of BING space, not used in the query.

Table S2. Oligonucleotide sequences of primers used in single-gene expression analysis by real-time RT-QPCR.

Gene name	Forward primer (5'-3')	Reverse primer (5'-3')
CXCL10	AAAGCAGTTAGCAAGGAAAG	TCATTGGTCACTTTTAGTG
CXCL11	TGCTACAGTTGTTCAAGGCTTCC	GGTACATTATGGAGGCTTTCTCAATATC
PODXL	CTACTAGACAGTGTTTCAC	GAGGTCTGTTGAGTTCTTTG
OAS1	ATTGTAAGAAGAAGCTTGGG	CAGAGTTGCTGGTAGTTTATG
GAPDH	ATCACCATCTTCCAGGAGC	CATGGTTCACACCCATGAC
Detection of the amplification products was carried out using SYBR Green I. The mRNA abundance of target genes in each cell line was normalized based on GAPDH abundance.		

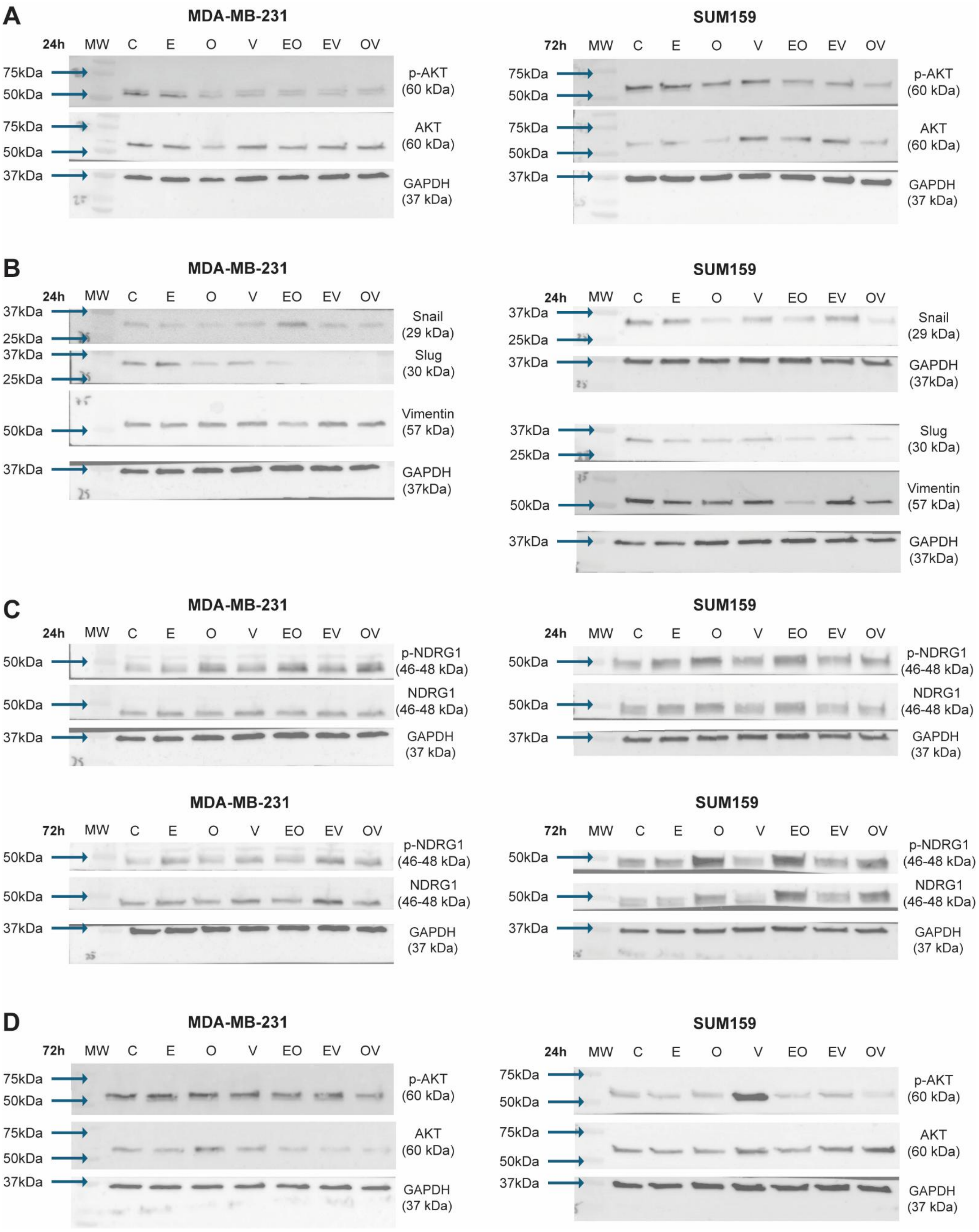
A
MDA-MB-231
24h MW C E O V EO EV OV
75kDa
50kDa
p-AKT (60 kDa)
75kDa
50kDa
AKT (60 kDa)
37kDa
GAPDH (37 kDa)
SUM159
72h MW C E O V EO EV OV
75kDa
50kDa
p-AKT (60 kDa)
75kDa
50kDa
AKT (60 kDa)
37kDa
GAPDH (37 kDa)
B
MDA-MB-231
24h MW C E O V EO EV OV
37kDa
25kDa
Snail (29 kDa)
37kDa
25kDa
Slug (30 kDa)
50kDa
Vimentin (57 kDa)
37kDa
GAPDH (37kDa)
SUM159
24h MW C E O V EO EV OV
37kDa
25kDa
Snail (29 kDa)
37kDa
GAPDH (37kDa)
37kDa
25kDa
Slug (30 kDa)
50kDa
Vimentin (57 kDa)
37kDa
GAPDH (37kDa)
C
MDA-MB-231
24h MW C E O V EO EV OV
50kDa
p-NDRG1 (46-48 kDa)
50kDa
NDRG1 (46-48 kDa)
37kDa
GAPDH (37 kDa)
SUM159
24h MW C E O V EO EV OV
50kDa
p-NDRG1 (46-48 kDa)
50kDa
NDRG1 (46-48 kDa)
37kDa
GAPDH (37 kDa)
MDA-MB-231
72h MW C E O V EO EV OV
50kDa
p-NDRG1 (46-48 kDa)
50kDa
NDRG1 (46-48 kDa)
37kDa
GAPDH (37 kDa)
SUM159
72h MW C E O V EO EV OV
50kDa
p-NDRG1 (46-48 kDa)
50kDa
NDRG1 (46-48 kDa)
37kDa
GAPDH (37 kDa)
D
MDA-MB-231
72h MW C E O V EO EV OV
75kDa
50kDa
p-AKT (60 kDa)
75kDa
50kDa
AKT (60 kDa)
37kDa
GAPDH (37 kDa)
SUM159
24h MW C E O V EO EV OV
75kDa
50kDa
p-AKT (60 kDa)
75kDa
50kDa
AKT (60 kDa)
37kDa
GAPDH (37 kDa)

107

Uncropped images of Western blots. (A) Membranes shown in Fig. 4B. (B) Membranes shown in Fig. 4C. (C) Membranes shown in Fig. S7A. (D) Membranes shown in Fig. S7B. C: vehicle control; E: efavirenz; EO: efavirenz + ouabain; EV: efavirenz + vinburnine; MW: molecular weight; O: ouabain; OV: ouabain + vinburnine, V: vinburnine.
